# Patient-Derived Organoid Serves as a Platform for Personalized Chemotherapy in Advanced Colorectal Cancer Patients

**DOI:** 10.3389/fonc.2022.883437

**Published:** 2022-06-01

**Authors:** Khamushavalli Geevimaan, Jing-You Guo, Chia-Ning Shen, Jeng-Kai Jiang, Cathy S. J. Fann, Ming-Jing Hwang, Jr-Wen Shui, Hsiu-Ting Lin, Mei-Jung Wang, Hsuan-Cheng Shih, Anna Fen-Yau Li, Shih-Ching Chang, Shung-Haur Yang, Jeou-Yuan Chen

**Affiliations:** ^1^ Institute of Biomedical Sciences, Academia Sinica, Taipei, Taiwan; ^2^ Genomic Research Center, Academia Sinica, Taipei, Taiwan; ^3^ Division of Colon and Rectal Surgery, Department of Surgery, Taipei Veterans General Hospital, Taipei, Taiwan; ^4^ Faculty of Medicine, School of Medicine, National Yang Ming Chiao Tung University, Hsinchu, Taiwan; ^5^ Department of Pathology, Taipei Veterans General Hospital, Taipei, Taiwan; ^6^ Department of Surgery, National Yang Ming Chiao Tung University Hospital, Yilan, Taiwan

**Keywords:** patient-derived organoids (PDOs), oxaliplatin sensitivity assessment, colorectal cancer, connectivity map, personalized chemotherapy

## Abstract

**Background:**

Addition of oxaliplatin to adjuvant 5-FU has significantly improved the disease-free survival and served as the first line adjuvant chemotherapy in advanced colorectal cancer (CRC) patients. However, a fraction of patients remains refractory to oxaliplatin-based treatment. It is urgent to establish a preclinical platform to predict the responsiveness toward oxaliplatin in CRC patients as well as to improve the efficacy in the resistant patients.

**Methods:**

A living biobank of organoid lines were established from advanced CRC patients. Oxaliplatin sensitivity was assessed in patient-derived tumor organoids (PDOs) *in vitro* and in PDO-xenografted tumors in mice. Based on *in vitro* oxaliplatin IC_50_ values, PDOs were classified into either oxaliplatin-resistant (OR) or oxaliplatin-sensitive (OS) PDOs. The outcomes of patients undergone oxaliplatin-based treatment was followed. RNA-sequencing and bioinformatics tools were performed for molecular profiling of OR and OS PDOs. Oxaliplatin response signatures were submitted to Connectivity Map algorithm to identify perturbagens that may antagonize oxaliplatin resistance.

**Results:**

Oxaliplatin sensitivity in PDOs was shown to correlate to oxaliplatin-mediated inhibition on PDO xenograft tumors in mice, and parallelled clinical outcomes of CRC patients who received FOLFOX treatment. Molecular profiling of transcriptomes revealed oxaliplatin-resistant and -sensitive PDOs as two separate entities, each being characterized with distinct hallmarks and gene sets. Using Leave-One-Out Cross Validation algorithm and Logistic Regression model, 18 gene signatures were identified as predictive biomarkers for oxaliplatin response. Candidate drugs identified by oxaliplatin response signature-based strategies, including inhibitors targeting c-ABL and Notch pathway, DNA/RNA synthesis inhibitors, and HDAC inhibitors, were demonstrated to potently and effectively increase oxaliplatin sensitivity in the resistant PDOs.

**Conclusions:**

PDOs are useful in informing decision-making on oxaliplatin-based chemotherapy and in designing personalized chemotherapy in CRC patients.

**Graphical:**
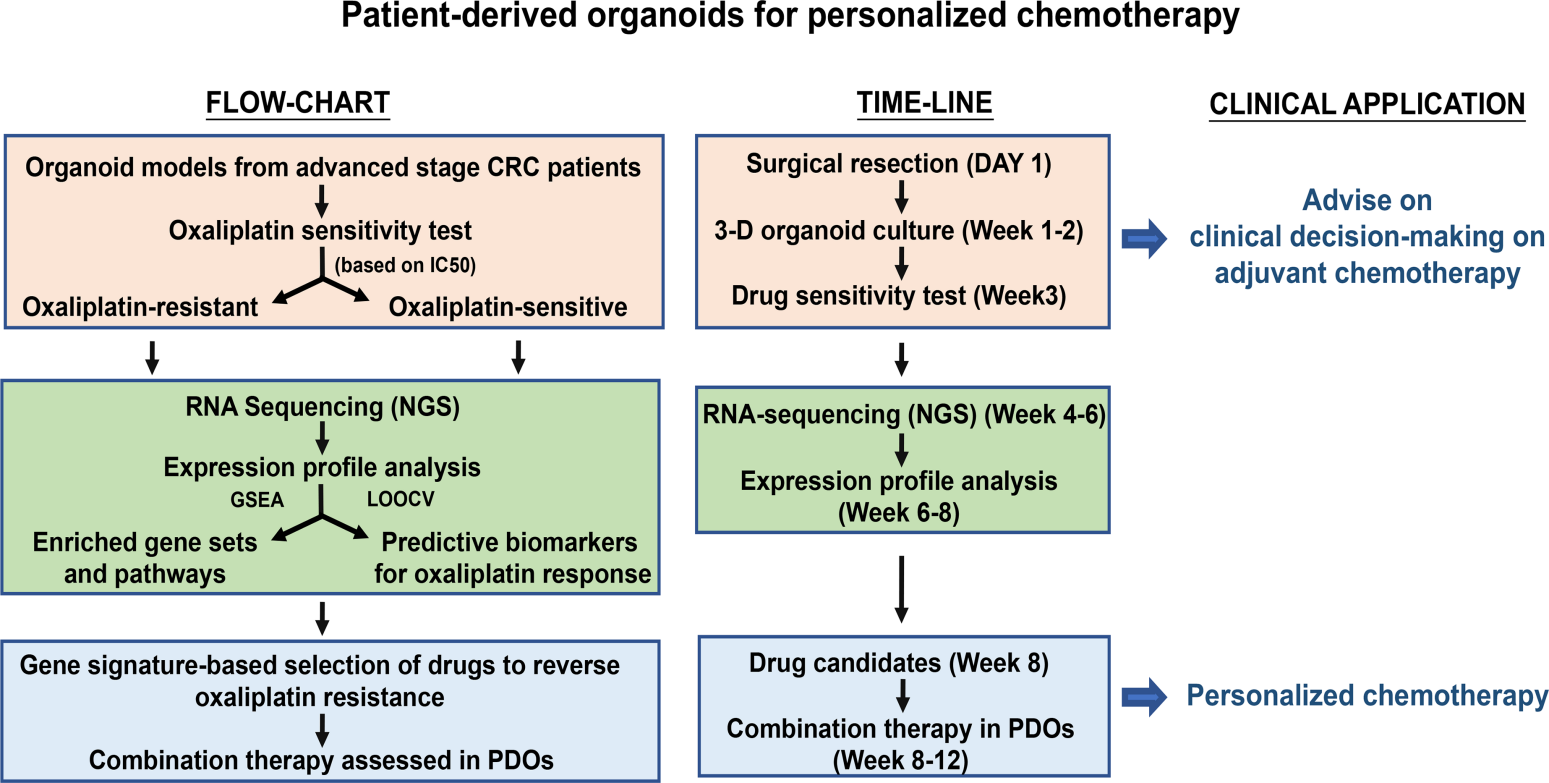


## Background

Colorectal cancer (CRC) is highly prevalent and accounts for ~500,000 deaths/year (1, 2). Although the advances in diagnosis and treatment modalities have led to reduced incidence and mortality, 50% of stage III and 95% of stage IV colorectal cancer patients succumb to this disease (American Cancer Society, 2011). Adding DNA-crosslinking agent oxaliplatin to antimetabolite drug 5fluorouracil (5FU), such as FOLFOX (leucovorin calcium (folinic acid), fluorouracil, and oxaliplatin) or CAPOX (capecitabine (Xeloda) and oxaliplatin), has yielded greater response rates and longer survival than with 5- FU alone in patients with high risk-stage II, stage III and metastatic CRC (3). However, response rates of oxaliplatin-based regimens only reach 40% - 45% (4). In addition, oxaliplatin-induced neuropathy has also prevented a subset of patients from receiving a complete course of oxaliplatin-based chemotherapy. Currently cancer treatment decisions are made based on clinical and pathologic staging and on molecular data. However, this method of prognosis does not predict drug response in individual patients. There is an urgent need to establish an *in vitro* model to predict patients’ response toward standard-of-care chemotherapy in advanced CRC, and to decide alternative treatment strategy for the resistant patients.

Recently, Sato et al. have established a novel *in vitro* 3D Intestinal Stem Culture (ISC) model by understanding the niche factor requirement for stem cell maintenance *in vivo* (5). This approach enables the growing of single ISCs to cyst-like with outward budding structure resembling intestinal epithelial crypt-villus organization, referred to as organoids. PDOs closely mimic the original primary tumor architecture and biology (6), and may overcome the limitations of traditional cell culture cancer models for *in vitro* drug assessment. As a support, a number of publications have demonstrated the feasibility of utilizing PDOs as a platform for prediction of patients’ response to therapy (7–13). Since oxaliplatin-based regimens serve as the first-line adjuvant therapies for CRC patients, in this study, we demonstrate the establishment of a living biobank of CRC patient-derived organoids and the usefulness of them as a predictive platform for the response of CRC patients to oxaliplatin-based chemotherapy. Predictive gene expression biomarkers are a promising and practical means for precision treatment. Taking drug response signature-based approaches, we have further identified compounds of great potential to increase oxaliplatin sensitivity in the resistant patients. Our findings demonstrate that PDOs may serve as an *in vitro* model to improve the precision and effectiveness of chemotherapy in advanced CRC patients.

## Materials and Methods

### Sample Collection,Organoid Preparation and Application

Fresh tissues and paraffin-embedded wax blocks were consecutively derived from patients who underwent surgical resection of CRC at Taipei Veterans General Hospital, Taiwan from February 13, 2017 to October 13, 2018. All studies were approved by the Institutional Review Board of Taipei Veterans General Hospital, Taiwan as well as the Institutional Review Board of Academia Sinica, Taiwan. Written consent forms were obtained from all the patients. In two patients, non-tumorous tissues were not obtained. Paired primary tumor tissues from the colon along with liver metastases were obtained from 3 patients. The average size of the tumor samples used to prepare PDO is 4-5 mm in diameter. Non-tumorous tissue was minced, digested with collagenase and dispase, and processed as described (14). The crypts were collected, embedded in matrigel, and cultured in Basal culture medium supplemented with 50% Wnt conditioned medium, 20% R-Spondin conditioned medium, 10% Noggin conditioned medium, 1x B27, 1.25 mM n-Acetyl Cysteine, 10 mM Nicotinamide, 50 ng/ml human EGF, 10 nM Gastrin, 500 nM A83-01, 3 M SB202190, 10 nM Prostaglandin E2, and 100 mg/ml Primocin (*In vivo*gen). Tumor tissue was minced, digested with Liberase, and processed as described previously (15). Tumor organoids were cultured in the same medium described above except without Wnt. Similarly, organoids were prepared from metastatic tumors from liver site in the same medium without Wnt, and adjacent non-tumorous liver tissues in media supplemented with 100 ng/ml FGF10 (Peprotech), 25 ng/ml HGF (Peprotech) and 10 μM Forskolin (Tocris) (16). In general, we established the organoids from the fresh tumor samples as passage 0. The organoids were passaged once in 6 days using TrypLE Express (Thermo Fisher Scientific) in 1:3 to 1:5 dilution based on cell density. After initial passages, the organoids (at passages 3 and 4) were frozen in a standard cell freezing media containing 10% dimethyl sulfoxide (DMSO) and stored at -80°C for 2 to 3 days and then shifted to liquid nitrogen tank for long term storage as organoid bio-bank.

In brief, we have established a bio-bank of 151 CRC-PDOs from 148 CRC patients, including 3 patients with matching liver metastatic tissues. The utilization of PDOs in this study is summarized. PDOs derived from advanced stages (III and IV) (n = 42) were subjected to *in vitro* drug sensitivity assay against oxaliplatin and divided into oxaliplatin-sensitive (OS) and -resistant (OR) groups. Four OR and 4 OS PDOs were used in xenograft tumor model study in mice for *in* *vivo* validation of the *in vitro* drug response. In addition, the drug responses in 17 PDOs derived from stage III (n = 12) and stage IV (n = 5) patients were further compared to the clinical outcomes of these patients after FOLFOX adjuvant therapy. To establish personalized medicine, 8 OS and 8 OR PDOs were subjected to RNA sequencing to build up molecular portraits of transcriptomes, followed by gene signature-based selection of candidate drugs.

### Immunofluorescence Analysis and Viability Assessment of Organoids

Whole mount immunofluorescence staining of organoids was performed as described earlier (17). Organoids were washed in ice-cold wash buffer (DMEM/F12 with 10% FBS), and the matrigel domes disrupted. Organoids were pelleted down and fixed with 4% paraformaldehyde in PBS for 30 min, and permeabilized with 0.5% Triton X-100 in PBS for 15 min. Organoids were washed in immunofluorescence (IF) wash buffer (0.1% BSA, 0.2% Triton X-100, 0.05% Tween 20), incubated with blocking buffer (1% BSA in IF wash buffer) for 1 h, and incubated with primary antibody rabbit anti-EpCAM (AbCam), rabbit anti-Mucin 2 (Santa Cruz), mouse anti-CK20 (Santa Cruz), or mouse anti-Chr-A (Santa Cruz) in blocking buffer for 2 h, followed by incubation with secondary antibody anti-mouse Alexafluor Texas Red (Invitrogen) or anti-rabbit or mouse Alexaflour 488 (Invitrogen) in blocking buffer for 1 h. After mounting in SlowFade anti-fade with DAPI (ThermoFisher), organoids were observed and micro graphed using Carl Zeiss LSM510 laser scanning microscope imaging system with Zen analysis software (Zeiss). Pathological sections of formalin-fixed paraffin-embedded patient tissue, xenografts and organoids were processed, imaged and analysed in the Histopathology unit at Institute of Biomedical Sciences, Academia Sinica. For organoids histology, whole organoids were pelleted down and fixed as described above. Then organoids were suspended in 2% low melting agarose, followed by dehydration, paraffin embedding, sectioning and Hematoxylin Eosin (H&E) staining.

### *In Vitro* Drug Sensitivity Assessment

Drug sensitivity assay was performed in organoids as described with modifications (14). Organoid cultures were gently disrupted into single cell suspension with TrypLE Express, and 3000 cells were seeded in 7 μl per well of matrigel diluted with equal volume of medium in 96-well plates in four replicates and cultured in organoid growth medium for 24 h. The cultures are incubated with oxaliplatin at various concentrations (0.03, 0.1, 0.3, 1, 3, 10, 30, and 100 μM) for 96 h, and subjected to CellTiter-Glo (Promega) cell viability assay. The slope of the dose-response curve was calculated. IC_50_ (half maximal inhibitory concentration) values are calculated with the four-parameter nonlinear logistic equation. E_max_ values were calculated as the percentage of inhibition at the maximum included concentration (100 μM). Based on IC_50_ values, PDOs were classified into four response categories, and the OR group includes the categories of non -responders and minor responders, and OS group include moderate and strong responders.

### RNA Sequencing

Total RNA was extracted from 8 OR and 8 OS PDO lines, using RNeasy Plus Mini Kit (QIAGEN). RNA-sequencing libraries were constructed using TruSeq Stranded mRNA Library Prep Kit (Illumina). The purified libraries were amplified by 10 cycles of PCR, and the resulted library profile was 250-500 bp, peaking at ~300 bps. Paired-end 2 x 101-nt sequencing was conducted on the Illumina HiSeq 2500 System (NGS High Throughput Genomics Core Facility at BRACS, Academia Sinica). The short-reads in FAST-Q format, ~85 M reads per sample, were processed by a computational pipeline as described (18). Briefly, the short reads were aligned and mapped to the Homo sapiens (human) genome assembly GRCh38 (hg38) using HISAT2. The aligned transcripts were assembled using StringTie by annotated GENCODE version 29. Gene expression levels were converted to counts by a python script (prepDE.py) provided by StringTie team. RNA-seq datasets consisted of 60,714 gene tags (25,213 Ensembl gene ids and 35,501 StringTie MSTRG ids) and 225,295 transcript tags (206,696 Ensembl transcript ids and 18,599 StringTie MSTRG ids), respectively, from 16 CRC PDOs. Fragments per kilobase of transcript per million mapped reads (FPKM) expression values from individual organoids were obtained. For comparison of differentially expressed genes (DEGs) and tags (DETs) between different groups, gene expression levels were further normalized by DESeq method and analyzed using Bioconductor package DESeq2.

### Molecular Profiling Using Bioinformatics Tools

The classification of the 16 PDOs was visualized by principal component analysis (PCA) (R package: ropls) and unsupervised hierarchical clustering analysis (Morpheus; Metric: One minus spearman rank correlation and Linkage method: average; https://software.broadinstitute.org/morpheus) using the DETs (fold change ≥ 2 and adjusted p-value < 0.05) as the identifier input. Analysis of the hallmarks and KEGG pathway enriched in the OR and OS PDOs were conducted by Gene Set Enrichment Analysis (GSEA). False discovery rate (FDR) q value < 0.05 was considered significant.

Leave-One-Out Cross Validation (LOOCV) was conducted to select oxaliplatin response gene predictors (19). Machine learning methods, such as Random Forest, K-Nearest Neighbors, Naïve Bayesian, Decision Trees, Neural Networks, Support Vector Machines, and Logistic Regression (LR), were applied to validate the prediction power of the sets of DEGs and DETs identified by LOOCV.

Connectivity MAP (C-MAP) online tool (https://www.broadinstitute.org/cmap/) was conducted to predict inhibitors targeting oxaliplatin resistant signatures (20, 21). Perturbagens ranked with the most high average negative enrichment scores and least variations were selected for further evaluation in combination therapy.

### Xenograft Studies

*In vivo* oxaliplatin response was assessed using PDO-based xenograft tumor model. All experimental protocols were approved by the Institutional Animal Care and Utilization Committee (IACUC) at Academia Sinica, Taiwan. Single cell suspension was prepared from each PDO, and 1×10^6^ cells were mixed with organoid growth medium/matrigel (1:1) in a total volume of 100 uL and injected subcutaneously to the right flank of NSG (NOD scid gamma) mice (8-9 weeks, 23-28 g). Tumors were allowed to grow to 80 ± 100 mm^3^ in size and randomly assigned into the control (2.5% DMSO/PBS) and treatment (oxaliplatin at 2 mg/kg in 2.5% DMSO/PBS) groups with 5 mice per group. Treatment was given by intra-peritoneal injection for 10 times at 3-day intervals. Tumor size and body weight were measured. TGI (Tumor Growth Inhibition) was determined at the end point of treatment period for each xenograft by the following formula: %TGI = {[1 – (T_t_/T_0_)/(C_t_/C_0_)]/[1 – (C_0_/C_t_)]} x 100, in which Tt and T0 were median tumor volumes of treated animal at time t and time 0, and C_t_ and C_0_ median tumor volumes of control group at time t and time 0, respectively (22). Median %TGI was calculated and reported for each group. Significant anti-tumor activity was defined as achievement of a median %TGI of at least 50%. Repeated two-way ANOVA was used to define the statistical difference between the groups using Graph Pad Prism 9.

### Statistical Analysis

Statistical analysis was performed in GraphPad Prism 9 (GraphPad Software, San Diego, CA). IC_50_ for *in vitro* drug assay was determined by non-linear regression log(inhibitor) vs response variable slope (four parameters). To calculate p-value in **Supplementary Figure 2**, Mann-Whitney test (un-paired two tailed) was used to compare the difference in IC_50_, E_max_, and AUC between the groups of minor/non-responders and moderate/responders. To calculate p-value in **Figure 3A**, we used Repeated Measure two-way ANOVA with the Geisser-Green house Correction and Bonferroni multiple comparison test. For all required studies, experimental mean was calculated and error was presented by standard deviation of the mean. For **Supplementary Figure S3**, significant difference of relapse-free survival depending on the PDO resistance or sensitivity to oxaliplatin was determined using Gehan-Breslow-Wilcoxon test. P<0.05 was considered as significant.

## Results

### Establishment of a Biobank of CRC Patient-Derived Organoids

**Figure 1:**
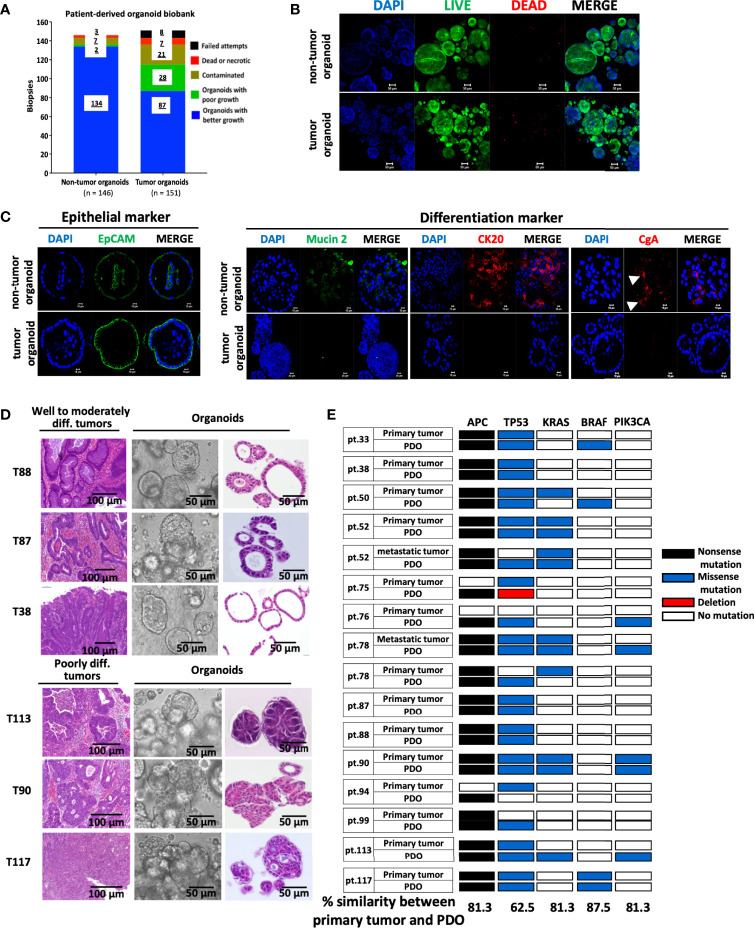
Establishment of a biobank of CRC patient-derived organoids. **(A)** Bar graph summarizes the preparation of organoid lines from the non-tumorous and tumorous tissues of 148 colorectal cancer patients. **(B)** The viability of organoids was assessed by immunofluorescence staining using LIVE/DEAD cell staining Kit. Confocal images of the live cells stained by Calcein-AM (in green), dead cells by EtBr (in red), and nuclei by DAPI (in blue), were shown. Scale bar, 50 m. **(C)** Confocal microscopy of the expression of EpCAM as epithelial marker, mucin 2 for Goblet cells, cytokeratin (CK) 20 for enterocytes, and chromagrannin (CgA) for neuroendocrine cells. **(D)** Histopathological features of primary tumors and PDOs. Representatives of bright-field images and H&E staining of tumor-derived organoids, and H&E staining of well to moderately differentiated (T88, T87 & T38) and poorly differentiated (T113, T90 & T117) tumors were shown. **(E)** Overview of mutational status in the driver genes in CRC primary/metastatic tumors and corresponding PDOs. The percentage of concordance between PDOs and original tumors in each driver gene is shown at the bottom.

Tumorous and non-tumorous tissues, plus liver metastases (n=3), were collected from 148 CRC patients, for organoid preparation. These CRC patients, categorized based on TNM staging, included 3 cases of stage 0, 24 of stage I, 42 of stage II, 57 of stage III, and 22 of stage IV, corresponding to 2%, 16.2%, 28.4%, 38.5% and 14.9%, respectively, of total samples collected. **Supplementary Table S1** lists the description of samples collected, and the 3 pairs of primary and metastatic tumor samples are indicated. The success rate of establishing organoid cultures was 93% (136 of 146) and 76% (115 of 151) for the non-tumorous and tumorous tissues, respectively (**Figure 1A**), which was comparable to previous reports (9, 23, 24). The unsuccessful cases included the initial attempts for optimization of culture conditions and those with tissues full of necrotic lesions or contaminated with pathogens. Among the established patient-derived tumor organoids (PDOs), 3 quarters of them, spanning stage 0 (n = 1), stage I (n = 13), stage II (n = 26), stage III (n = 30), and stage IV (n = 17), were characterized with good growing condition, and can be expanded for continuous passages (passage number > 10). Wnt3A was required for culturing organoids derived from non-tumorous tissues, but not for most, if not all, of the tumor-derived organoids. Non-tumor organoids can only be cultured and maintained under normoxic condition, whereas tumor organoids were able to proliferate under both normoxic and hypoxic conditions. We routinely cultured tumor organoids under normoxia in the absence of Wnt3A. Immunofluorescence staining was performed to show the viability of the organoids (**Figure 1B**). Almost all the cells in both non-tumor and tumor organoids were stained positively by the live-cell-permeable green fluorescent dye Calcein-AM. The organoids were stained positive for the epithelial cell adhesion molecule (EpCAM). The non-tumor organoids were readily stained for mucin 2, cytokeratin-20 and chromagranin A, demonstrating the presence of differentiated cell types of intestinal epithelial lineage, whereas most of the tumor organoids were stained weakly or negative for the differentiation markers (**Figure 1C**). CRC-derived organoids cultured under *in vitro* or *in vivo* xenograft condition retained architectural features resembling the tumor tissues from which they were derived (**Supplementary Figure S1**). Well and moderately differentiated tumors gave rise to cyst-like organoids with clear lumen formation, whereas the organoids derived from poorly differentiated tumors were characterized with highly compact structures (**Figure 1D**). Sequencing analyses revealed that PDOs preserved most of the mutational status of the driver genes in the primary tumors (**Figure 1E**). We noted that there were mutations either gained or lost in a small number of PDO lines during the *in vitro* culturing process.

### Assessment of Oxaliplatin Response in Advanced CRC PDOs

**Figure 2:**
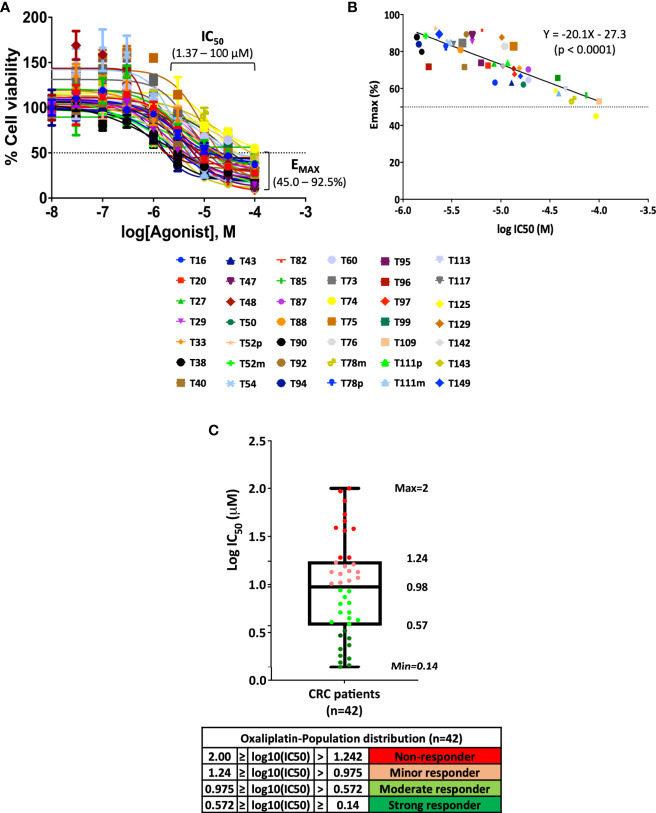
Pattern of oxaliplatin response in 42 CRC-derived organoid models. **(A)** Fitted dose response curves for oxaliplatin. Each curve represents the mean and standard deviation of four replicates per condition. **(B)** Linear regression curve between potency (IC_50_) and efficacy (E_max_) of 42 PDOs treated with oxaliplatin (p <0.0001). **(C)** Box plot using log_10_IC_50_ to define drug response of 42 CRC-PDOs into the categories of strong responders, moderate responders, minor responders, and non-responders towards oxaliplatin treatment.

Adjuvant 5-FU in combination with oxaliplatin serves as the first-line chemotherapy for patients of advanced CRC (25, 26). With the aim to address whether PDO lines can serve as a preclinical model to guide therapeutic decisions for advanced CRC patients, we performed drug sensitivity assessment of advanced CRC PDOs towards oxaliplatin. Forty-two of the 47 advanced CRC PDOs were successfully recovered from frozen stocks without showing any decline in growth rate, and subjected to the treatment of increasing concentrations of oxaliplatin. The dose response curve of individual PDOs was constructed (**Figure 2A**). The IC_50_ ranged from 1.37 to 100 μM, varying in 2 orders of magnitude in individual PDOs, and E_max_ ranged from 45 to 93%. An inverse correlation was established between the potency and efficacy of oxaliplatin in the 42 PDOs (**Figure 2B**). The wide range of differences in the potency and efficacy of oxaliplatin emphasizes the need for personalized medicine. According to IC_50_, the PDOs were grouped into four categories (**Figure 2C**), strong responders (log_10_IC_50_ 0.14 – 0.57), moderate responders (log_10_IC_50_ 0.57 – 0.98), minor responders (log_10_IC_50_ 0.98 – 1.24), and non-responders (log_10_IC_50_ 1.24 – 2.00), as previously described^6^. Strong and moderate responders were defined as oxaliplatin-sensitive (OS) group whereas minor and non-responders were defined as oxaliplatin-resistant (OR) PDOs. Individual dose response curves of oxaliplatin-sensitive and -resistant PDOs are presented in **Supplementary Figure S2A**. Quantification of responses to oxaliplatin by calculating the IC_50_, E_max_ and the area under the dose response curve (AUC_DRC_) showed a significant difference between the resistant (non-responders/minor responders) and sensitive (moderate/strong responders) groups (**Supplementary Figure S2B**). **Supplementary Table S2** summarizes the clinical data of the 42 advanced CRC PDOs, along with their *in vitro* oxaliplatin response.

### *In Vivo* Validation of Oxaliplatin Response

**Figure 3:**
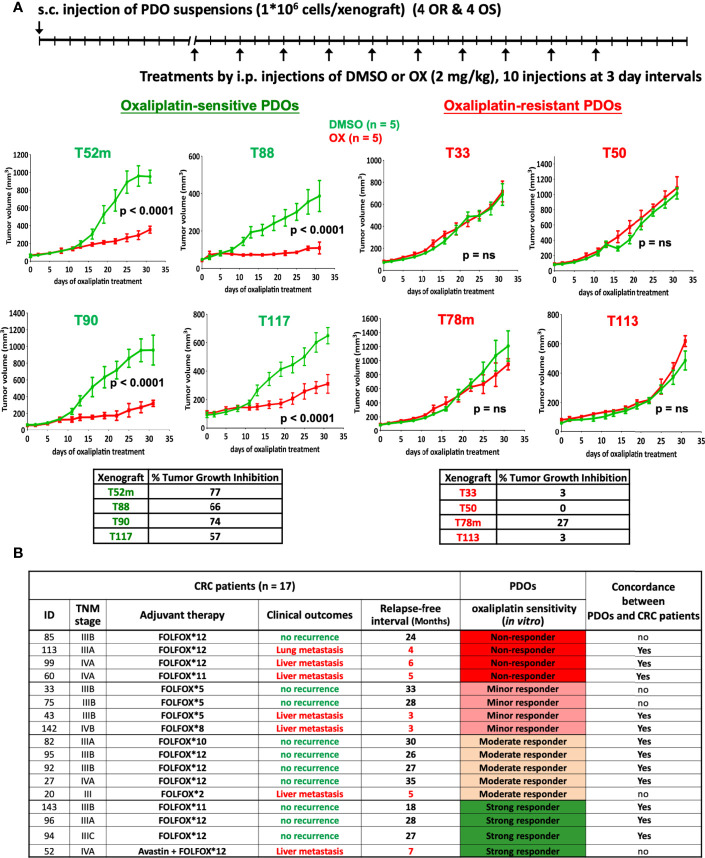
*In vivo* validation of oxaliplatin response. **(A)** Oxaliplatin sensitivity in PDO xenograft tumor model. Suspensions of OR (T33, T50, T78m, and T113) and OS (T52m, T88, T90, and T117) PDOs were subcutaneously transplanted into NSG mice, and treated by intraperitoneal injection with vehicle (2.5% DMSO) or oxaliplatin (2 mg/kg) in 5 mice each group for 10 injections. Tumor volume was monitored. Oxaliplatin-mediated inhibition on tumor growth was calculated. The p-value was obtained by using Repeated Measure two-way ANOVA with the Geisser-Green house Correction and Bonferroni multiple comparison test. P < 0.01 was considered as significant. **(B)** Oxaliplatin sensitivity test in PDOs predicts FOLFOX treatment outcomes in CRC patients. Patients’ ID, TNM stage, and the course and outcome of FOLFOX adjuvant therapy are listed. CRC patients with no-recurrence are considered as responding to FOLFOX treatment whereas patients with lung or liver metastasis recurrence as non-responding to FOLFOX. Concordance between oxaliplatin response in PDOs and patients is shown as yes, whereas discordance as no.

We next examined whether the oxaliplatin sensitivity determined in PDOs *in vitro* can be validated *in vivo*. As shown in **Figure 3A**, oxaliplatin-resistant (OR) and -sensitive (OS) PDOs were subjected to xenograft tumor model in NSG mice, followed by oxaliplatin treatment. Oxaliplatin-mediated tumor growth inhibition (TGI) was calculated as described (22). As shown, oxaliplatin treatment yielded significant inhibition (%TGI > 50%) in all of the 4 OS PDO xenografts. To the contrast, none of the OR PDO xenografts responded well to oxaliplatin-mediated inhibition. These results demonstrate a good concordance between the oxaliplatin sensitivity in PDOs and drug response in PDO xenograft tumors *in vivo*.

Next, we analyzed the clinical outcomes of CRC patients who had received oxaliplatin-based therapy. Among the 42 advanced CRC PDOs, 17 were derived from patients (12 of stage III and 5 of stage IV) who underwent FOLFOX adjuvant therapy. **Supplementary Table S3** describes the details of the patient cohort from whom these 17 PDOs were obtained. According to oxaliplatin sensitivity assessed *in vitro*, the 17 PDOs were 4 non-responders, 4 minor responders, 5 moderate responders, and 4 strong responders to oxaliplatin. Notably, the patients who developed lung or liver metastasis after adjuvant therapy belonged to 3 of the 4 non-responders, 2 of the 4 minor responders, 1 of the 5 moderate responders, and 1 of the 4 strong responders (**Figure 3B**), demonstrating that FOLFOX treatment response in advanced CRC patients followed the trend of oxaliplatin sensitivity determined in PDOs (P = 0.047, using single tailed Cochran-Armitage trend test). Further, Kaplan Meier relapse-free curve of CRC patients depending on PDO resistance or sensitivity to oxaliplatin showed a significant difference (P = 0.047, using Gehan-Breslow-Wilcoxon test) (**Supplementary Figure S3**). To summarize clinical validity of PDO-based drug screen results for predicting treatment response, we found 70% sensitivity, 71.4% specificity, 77.8% positive predivtive value, and 62.5% negative predictive value in predicting response to oxaliplatin-based regimen (Fisher’s exact test p = 0.15).

### Molecular Portraits of Oxaliplatin Response in Advanced CRC PDOs

**Figure 4:**
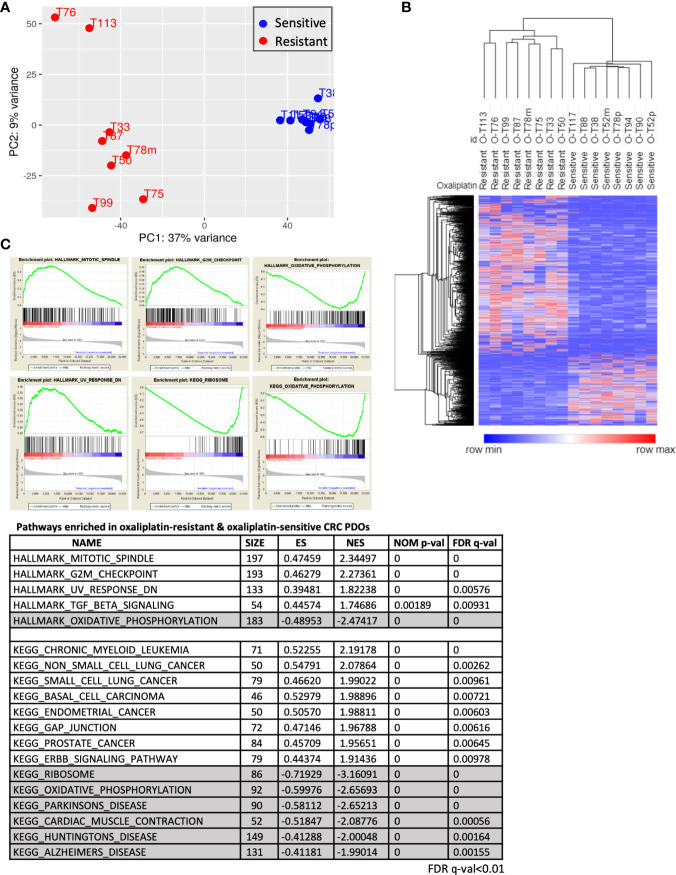
Molecular portraits of oxaliplatin response in advanced CRC PDOs. **(A)** Principal component analysis, using the 2555 DETs as input, assesses the variance of the 16 PDOs. The OS and OR PDOs are depicted in red and blue, respectively. **(B)** Hierarchical clustering analyses performed using DETs between OS and OR PDOs. In hierarchical clustering, column represents sample and row represents transcript. Expression values (FPKM) were depicted from high (in red) to low (in blue). **(C)** Hallmarks and KEGG pathways (FDR q-value < 0.01) enriched in OR and OS PDOs based on GSEA algorithm. Hallmarks and pathways with positive normalized enrichment score (NES) indicate positive correlation with OR phenotype, and negative NES value with OS phenotype.

To investigate the molecular mechanisms modulating oxaliplatin response, we analyzed the expression profiles of 8 OS and 8 OR PDOs. RNA sequencing was performed. The short reads in Fast-Q format were processed through the pipeline of HISAT2, StringTie and DESeq2 algorithms, and 2555 differentially expressed transcripts (DETs, adjusted p < 0.05 and fold change ≥ 2) were obtained. By principal component analysis and unsupervised hierarchical clustering analysis (**Figures 4A, B**), these DETs yielded a clear separation between the OS and OR PDOs. Morpheus Marker selection method (https://software.broadinstitute.org/morpheus) was applied, using a permutation test with 1,000 replications, and transcripts correlated to the OR and OS phenotypes were also identified. **Supplementary Figure S4** shows the heatmap of top 25 up- and 25 down-regulated transcripts associated with the OR phenotype in the form of hierarchical clustering (Spearman’s correlation), demonstrating again that OS and OR PDOs exhibited distinct expression profiles.

**Figure 5:**
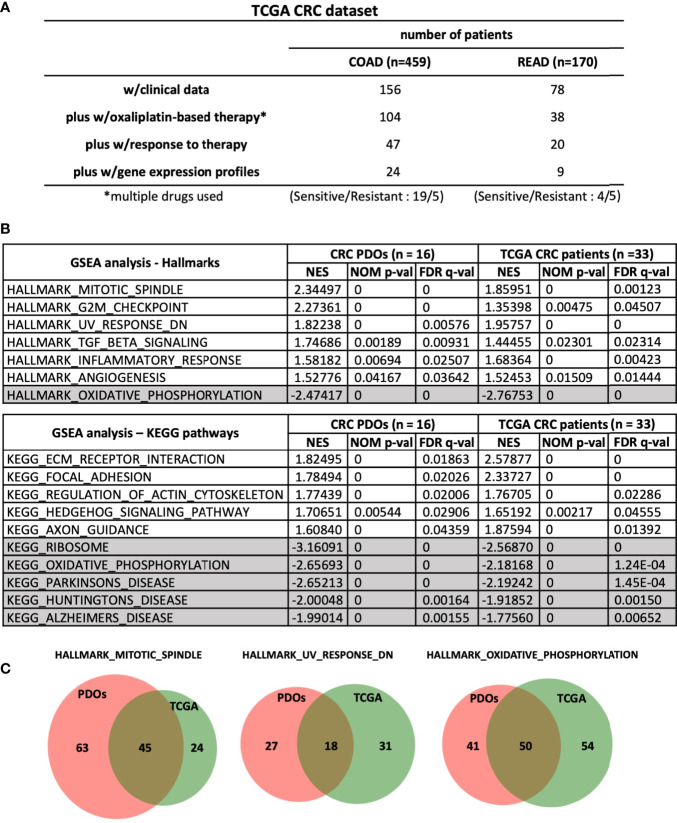
Common pathways and gene sets are associated with oxaliplatin responses in CRC PDOs and patients. **(A)** Stratification of patients in TCGA-CRC dataset. **(B)** Hallmarks and pathways associated with OR and OS PDOs as well as with CRC patients (n = 24 for COAD and n = 9 for READ) responding and non-responding to FOLFOX treatment. NES, normalized enrichment score. NOM p-val, nominal p value. FDR q-val, false discovery rate q-value. **(C)** Venn-diagrams showing the gene sets shared between CRC-PDOs and TCGA-CRC patients in the hallmarks enriched in OR and OS phenotypes. Numbers of genes were depicted. *, means - CRC patients were treated with oxaliplation in combination with other drugs. So we labelled it as multiple drugs used.

By Gene Set Enrichment Analysis (GSEA) (www.broad.mit.edu/gsea), hallmarks of mitotic spindle, G2/M checkpoint, UV response and TGF-β signaling were identified to be tightly associated with OR phenotype, whereas oxidative phosphorylation hallmark associated with OS phenotype (**Figure 4C**). To validate our findings, we further queried RNA-sequencing data of human CRC (459 of COAD and 170 of READ) database in The Cancer Genome Atlas (TCGA) program. Among the patients who received oxaliplatin-based chemotherapies with clinical outcomes recorded, 33 were evaluable for having RNA-Seq data (**Figure 5A**). According to treatment response, 23 patients were considered to be resistant to the treatment for developed stable disease (SD) or progressive disease (PD), whereas 10 exhibited complete response (CR) or partial response (PR) as responding or sensitive to the treatment. By GSEA analysis, the pathways and gene sets associated with CRC patients resistant and sensitive to oxaliplatin-based treatments were shown to be similar to those enriched in the OR and OS PDOs (**Figures 5B, C** and **Supplementary Figure S5**).

### Identification of Oxaliplatin Response Predictor Signatures

**Figure 6:**
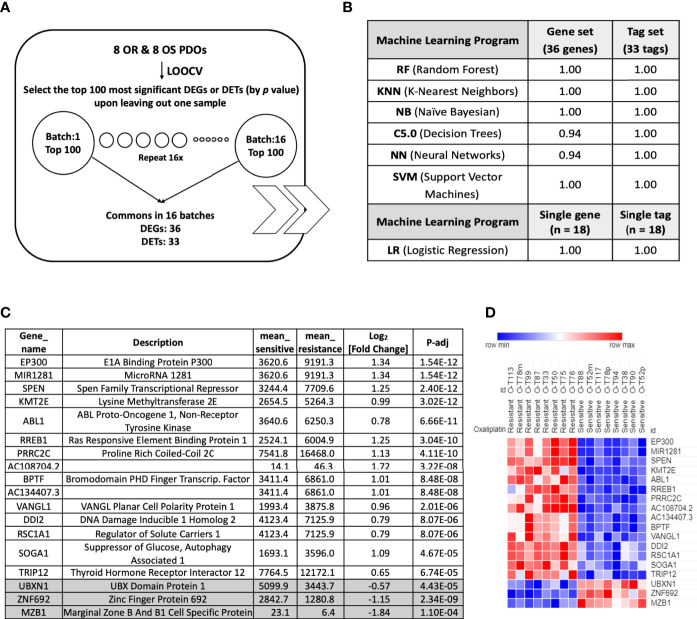
Oxaliplatin response predictor genes identified by Leave-One-Out Cross Validation (LOOCV) algorithm. **(A)** Workflow of identification of oxaliplatin response signatures using LOOCV algorithm to compare genes and transcripts differentially expressed between OR and OS PDOs. **(B)** Machine learning algorithms to evaluate the accuracy of predictor response genes and transcripts with their scores. **(C)** Oxaliplatin response predictor genes identified by LOOCV algorithm, along with their mean expression values in the OR and OS PDOs and log_2_[fold change] with adjusted P < 0.005. **(D)** Heat map of oxaliplatin response predictor genes identified by LOOCV algorithm.

To identify oxaliplatin response predictor signatures, Leave-One-Out Cross Validation (LOOCV) algorithm was performed to compare the expression profiles of 8 OR vs. 8 OS PDOs (**Figure 6A**). Sixteen new expression datasets were created by alternatively leaving out the RNA-Sequencing dataset of one PDO. Comparative analysis was performed in each dataset, and the top 100 DEGs and DETs between the OR and OS PDOs were identified. Among the 16 sets of top 100 DEGs and DETs, 36 DEGs and 33 DETs were found commonly present in all the 16 sets of genes and transcripts. Several machine learning programs were applied to evaluate the accuracy of using the sets of 36 DEGs and 33 DETs as predictors for oxaliplatin response. As shown, the set of 36 DEGs or 33 DETs gave a perfect or near perfect value of 1 as prediction accuracy to serve as drug response predictor signatures. We further exercised to test whether any single gene in these DEGs and DETs can serve as a drug response predictor. Using Logistic Regression (LR) model, we identified 18 models using one single gene and 20 models using one single transcript that reached the score of 1 to predict oxaliplatin response in the PDOs (**Figure 6B**). The identity of the 18 oxaliplatin response predictor signatures and their expression status in OR and OS were shown (**Figures 6C, D**). These predictor signatures also fell within the most significantly differentially expressed genes identified by GSEA (**Supplementary Table S4**).

### Gene Signature-Based Approaches to Identify Candidate Drugs to Sensitize Oxaliplatin Response

By GSEA, hallmarks of mitotic spindle and G2/M checkpoint were identified to be tightly associated with the OR phenotype. **Supplementary Table S5** lists the gene sets associated with these two hallmarks that were enriched in the OR phenotype, and a cohort of genes were commonly enriched in these two hallmarks, including c-ABL and NOTCH2. We hypothesized that inhibition of these two hallmarks may attenuate oxaliplatin resistance. Small molecules of c-ABL inhibitor imatinib and DAPT which targets NOTCH pathway by inhibiting γ- secretase were chosen to be tested.

**Figure 7:**
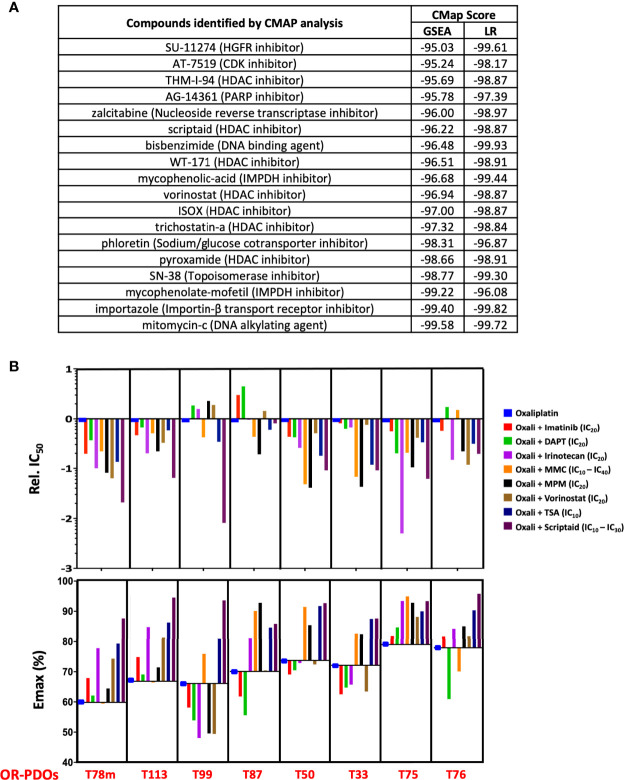
Drug response signature-based combination chemotherapy. **(A)** Connectivity-MAP analysis identifies potential therapeutic drugs targeting OR signatures in PDO’s. Enrichment scores from searches using gene sets identified by GSEA and LOOCV/LR models are shown. **(B)** Response to oxaliplatin-based combination therapy in OR PDOs. Bar graphs show the IC_50_ and Emax of each PDO towards oxaliplatin alone or in combination with imatinib, DAPT, irinotecan, mitomycin-C, mycophenolate mofetil, vorinostat, trichostatin-A, or Scriptaid at designated dosage. IC_50_ is shown in relation to the IC_50_ of oxaliplatin alone. For MMC and scriptaid, IC_10_ was used for PDOs which were particularly sensitive to the drugs, and IC_30_ or IC_40_ were used for the PDOs less sensitive to the drugs.

In a second approach, we submitted the top 150 up-regulated and 150 down-regulated genes associated with OR phenotype by GSEA to the Connectivity Map (CMAP) (20), a genomics-based drug discovery framework, and identified 24 perturbagens with enrichment scores of ≤ -95, implicating that they may target OR signatures. Similarly, the 15 predictor genes derived from LOOCV that were up-regulated in the OR PDOs were also subjected to C-MAP analysis, and 128 compounds with enrichment scores ≤ -95 were identified. Eighteen compounds were found commonly shared in these two groups of perturbagens, including HDAC inhibitors, DNA damaging agents, DNA/RNA synthesis inhibitor, and inhibitors for druggable targets, such as CDK inhibitor and HGFR inhibitor (**Figure 7A**). Six compounds with relatively high negative enrichment scores, including irinotecan, mitomycin-c, HDAC inhibitors vorinostat, scriptaid, and trichostatin A (TSA), and mycophenolate-mofetil (MPM), a selective inhibitor of inosine monophosphate dehydrogenase that may inhibit DNA/RNA synthesis, were chosen to be tested for their activity to increase oxaliplatin sensitivity. Cytotoxicity of these compounds in PDOs was assessed, and a minimum effective dose, between IC_10_ – IC_40_, was chosen for each compound in combination therapy with oxaliplatin. Dose response curve for oxaliplatin alone and in combination with individual compounds was constructed, and relative IC_50_ of oxaliplatin resulted from the combination therapy and the Emax achieved in OR PDOs were shown in **Figure 7B**. In comparison to oxaliplatin monotherapy, most of the combination therapies significantly increased the potency and efficacy of oxaliplatin, as evidenced by the decrease of relative IC_50_ by 1 to 2 orders of magnitude (top panel), and the increase of E_max_ by 10 - 20% (bottom panel). Since these candidate drugs were selected based on their potential to target OR phenotype, as expected, favorable outcomes were not expected when treating the OS PDOs (**Supplementary Figure S6**). Notably, a much worse effect was observed when applying the combination therapies to the OS PDOs, as evidenced by the dramatically increased IC_50_ and decreased E_max_. **Supplementary Table S6** summarizes the IC_50_ values of individual compounds and in combination with oxaliplatin. These data further emphasize the importance of gene signature-based approach for personalized therapy. Most importantly, these data strongly support the usefulness of PDOs as a platform for the development of gene-based therapies.

## Discussion

Systemic chemotherapy remains as the cornerstone in the management of CRC patients. There is an urgent need to establish a pre-clinical model that can predict patients’ response toward chemotherapy. In this study, we established a biobank of CRC PDOs with the aim to assess the usefulness of PDOs in predicting the response of advanced CRC patients towards oxaliplatin-based chemotherapy. Paired non-tumorous and tumorous tissues were recruited from 148 colorectal cancer patients for establishing organoid lines, and the success rate was 93% and 76%, respectively. The patient-derived CRC organoid cultures recapitulated pathological and molecular features of the tumors from which they were derived, suggesting PDOs as a useful platform for drug testing, drug discovery and disease modeling. Dose response curve for oxaliplatin was constructed and oxaliplatin sensitivity assessed in 42 advanced CRC-PDOs. The oxaliplatin response assessed in PDO lines was further validated *in vivo*. We showed that oxaliplatin-mediated inhibition on tumor growth in PDO xenograft tumor model in mice correlated to the oxaliplatin sensitivity assessed in PDOs. Furthermore, the outcomes of CRC patients undergone FOLFOX treatment were shown followed the trend of oxaliplatin sensitivity assessed in PDOs. These results demonstrate that PDOs may serve as a platform to predict patients’ response to oxaliplatin treatment, and thus providing information to advise clinical decision-making for adjuvant therapy. Most importantly, PDO test allows to identify the subset of advanced CRC patients who may not benefit from FOLFOX treatment, and thus prevents them from unnecessary and ineffective treatment.

With the aim to increase oxaliplatin treatment response in the resistant patients, we painted molecular portraits of OR and OS phenotypes. RNA sequencing was performed and bioinformatics analysis (PCA and hierarchical clustering) revealed a clear separation between the OR and OS PDOs, suggesting that oxaliplatin responsiveness is undermined by specific molecular mechanisms. In support, GSEA showed that OR phenotype was associated with the hallmarks of mitotic spindle, G2/M and others, whereas OS phenotype was associated with hallmark of oxidative phosphorylation. These findings were in lines with previous reports that oxaliplatin killed cells by inducing ribosome biogenesis stress (18) and knockdown of genes involved in mitotic spindle and G2/M checkpoint, such as BRCA2, efficiently sensitized cancer cells to oxaliplatin treatment (27). We further confirmed our findings by analyzing the dataset of human colorectal adenocarcinoma patients in TCGA database and demonstrating that similar hallmarks and gene sets were also identified in colorectal adenocarcinoma patients who were resistant and sensitive to oxaliplatin-based chemotherapy, respectively. Taken together, these results highlighted the potential pathways that may dominate oxaliplatin responsiveness, and would assist to develop strategies that may overcome oxaliplatin resistance in CRC patients.

Under the notion that OR and OS phenotypes were each characterized with distinct hallmarks and gene sets, we further identified 18 biomarker signatures that are predictive of oxaliplatin responsiveness using LOOCV algorithm followed by Logistic Regression model. Next, utilizing PDOs as an *in vitro* model, we took gene-based approach to develop personalized chemotherapy. In order to suppress OR phenotype, we submitted the OR phenotype-associated gene signatures to CMAP algorithm, and identified perturbagens with the most negative enrichment scores, including DNA damaging agents, DNA/RNA synthesis inhibitor, HDAC inhibitors, tyrosine kinase inhibitors, and etc. Several of them have already been in clinical use like topoisomerase inhibitor irinotecan (28), mitomycin C (29), and HDAC inhibitor vorinostat (30, 31), and in clinical trials like mycophenolic-acid (dehydrogenase inhibitor) (32), or have been shown to have anti-tumor activity *in vitro* and *in vivo* like HDAC inhibitors trichostain-A (33, 34) and scriptaid (35–37). Interestingly, HDAC inhibitors have also been shown to exhibit additive or synergistic activity with oxaliplatin to sensitize cell lines derived from CRC (38) and other types of cancer (39). Six of the perturbagens, including irinotecan, mitomycin C, MPM, vorinostat, TSA, and scriptaid were chosen to be combined with oxaliplatin and tested for their effectiveness in increasing oxaliplatin sensitivity. In addition, inhibitors to c-ABL and γ-secretase were also chosen for targeting the core components of mitotic spindle and G2/M pathways, the major pathways associated with oxaliplatin-resistant phenotype. NOTCH-ABL axis has been implicated in colorectal cancer metastasis (40). Our data showed that these 6 perturbagens plus c-ABL inhibitor and γ-secretase inhibitor can significantly increase the potency and efficacy of oxaliplatin upon treating the OR PDOs, including the PDO derived from liver metastasis, suggesting the feasibility of gene signature-based therapeutic strategy. These candidate drugs were chosen based on their potential to antagonize OR phenotype, as expected, they failed to facilitate but rather impeded oxaliplatin-mediated cytotoxicity in the OS PDOs, emphasizing the importance of personalized medicine.

In this study, oxaliplatin sensitivity was assessed in 42 advanced CRC PDOs, including 3 matching sets from the primary tumors and their liver metastases. Increased drug resistance was observed in the metastatic tumor-derived organoids as compared to the primary tumor-derived organoids (T78p vs T78m and T111p vs T111m) (**Table S2**), consistent to the notion that accumulated genetic alterations drive disease progression. The remaining set (T52p and T52m) displayed similar oxaliplatin sensitivity, however, different drug responses towards combination therapies implicated that they harbored different genetic alterations (**Supplementary Figure S6**). Nevertheless, we demonstrated that gene-based formulation of combination therapy yielded improved therapeutic efficacy in both the PDOs derived from primary as well as metastatic tumors (data on T78m were shown). In this study, although the success rate of establishing PDOs was high, about 25% of CRC-PDOs displayed sub-optimal growth condition and drug sensitivity test cannot be properly assessed. In addition, we also suffered 10% loss of the organoid lines through freezing and thawing process. Improved culture conditions are necessary, so PDO-informed drug assessment can be universally applied to most if not all of the patients.

Several studies have demonstrated the potential usage of PDOs in guiding patients’ treatment (8–12), including a prospective study reported by Ooft et al., which demonstrated that PDO test predicted response of more than 80% of metastatic CRC patients treated with 5-FU-irinotecan but not 5-FU-oxaliplatin combination therapy (10). In our study, we evaluated the usefulness of oxaliplatin response in advanced CRC PDOs (n = 17) in predicting clinical outcome of patients receiving FOLFOX adjuvant therapy. Among the 9 patients with their PDOs being sensitive to oxaliplatin, 7 remained no recurrence, giving a 78% prediction rate. As to the other 8 patients whose PDOs displaying resistant phenotype, as minor or non-responders to oxaliplatin, 5 developed recurrent or progressive disease after FOLFOX treatment, giving a 63% prediction rate. Thus, oxaliplatin sensitivity test in PDOs yielded a 70.6% (12 out of 17) accuracy in predicting patients’ clinical outcome towards FOLFOX adjuvant therapy, demonstrating the usefulness of PDO as an *in vitro* model to assist clinical decision-making on first-line adjuvant chemotherapy for advanced CRC patients. The discrepancy on PDO test to predict oxaliplatin sensitivity between the study of Ooft et al. and ours may lie in the difference in patients’ groups and other factors. Since both studies were based on patients of a small number, a clinical study including larger case number is underway to further evaluate whether PDOs can predict the response toward oxaliplatin-based treatment in advanced stage CRC patients.

## Conclusion

This study shows that oxaliplatin sensitivity assessed in advanced CRC patient-derived organoids was correlated to patients’ response to FOLFOX treatment, and drugs identified by gene signature-based approach significantly improved oxaliplatin sensitivity for personalized treatment, suggesting PDOs as a useful platform to inform clinical decision-making on adjuvant chemotherapy and in designing personalized chemotherapy.

## Data Availability Statement

The datasets presented in this study can be found in online repositories. The names of the repository/repositories and accession number(s) can be found below: BioProject, accession number PRJNA814344, and BioSample, accession numbers SAMN26549552-SAMN26549567.

## Ethics Statement

The studies involving human participants were reviewed and approved by Institutional Review Board of Taipei Veterans General Hospital, Taiwan as well as the Institutional Review Board of Academia Sinica, Taiwan. The patients/participants provided their written informed consent to participate in this study.

## Author Contributions

GK, J-YG, S-HY, and J-YC designed the research, GK, J-YG, C-NS, H-TL, M-JW, J-WS, and AF-YL performed experiments, J-KJ, S-CC, and S-HY performed clinical studies, CF, M-JH, and H-CS carried out computational analyses, GK and J-YC wrote the manuscript. GK and J-YG equally contributed to the manuscript. Correspondence and requests for materials should be addressed to S-HY and J-YC. All authors approved the manuscript.

## Funding

This work was supported by the Taiwan Ministry of Sciences and Technology Research Project Grants (106-2321-B-400-014 and 107-2314-B-400-016 to CN Shen, SH Yang, JY Chen; 106-2314-B-075-079 and 107-2314-B-010-067-MY2 to SH Yang), and the Key and Novel Therapeutics Development Program for Major Diseases project of Academia Sinica, Taiwan, R.O.C. (AS-KPQ-111-KNT to CN Shen).

## Conflict of Interest

The authors declare that the research was conducted in the absence of any commercial or financial relationships that could be construed as a potential conflict of interest.

## Publisher’s Note

All claims expressed in this article are solely those of the authors and do not necessarily represent those of their affiliated organizations, or those of the publisher, the editors and the reviewers. Any product that may be evaluated in this article, or claim that may be made by its manufacturer, is not guaranteed or endorsed by the publisher.

## Supplementary Material

Supplementary Figures and Tables.

Supplementary Figure 1 H&E staining of primary tumors, tumor-derived organoids, and PDO xenografts in NGS mice. Scale bars: tumor tissues, 100 μm; tumor organoids, 50 μm; PDO xenograft, 100 μm.

Supplementary Figure 2
**(A)** Fitted dose response curves for oxaliplatin-resistant and -sensitive PDOs. Each curve represents the mean and standard deviation of four replicates per condition. **(B)** Quantification of oxaliplatin response using IC_50_, E_max_ and AUC between the sensitive (strong/moderate responders) and the resistant (minor/non-responders) PDOs. Comparison was made using a two-tailed Mann-Whitney test (p < 0.0001). Dots represent individual PDOs, horizontal bars represent the mean, and error bars indicate SD.

Supplementary Figure 3 Kaplan Meier relapse-free curve of CRC patients depending on PDO resistance or sensitivity to oxaliplatin. Comparison was made using Gehan-Breslow-Wilcoxon test (p = 0.047). This plot is based upon Fig.3B, where recurrence is given as value 1 and non-recurrence as 0.

Supplementary Figure 4 Heatmap of top 25 up- and 25 down-regulated transcripts associated with the OR phenotype in the form of Hierarchical clustering (Spearman’s correlation) by Morpheus Marker selection method (Input: 2555 differential expressed transcripts; 1000 permutations).

Supplementary Figure 5 Complete list of significant hallmarks and pathways that are upregulated in oxaliplatin-resistant and -sensitive TCGA CRC patients and CRC-PDOs.

Supplementary Figure 6 Response to oxaliplatin-based combination therapy in OS PDOs. Bar graphs show the IC_50_ and E_max_ of each PDO towards oxaliplatin alone or in combination with imatinib, DAPT, irinotecan, mitomycin-C, mycophenolate mofetil, vorinostat, trichostatin-A, or Scriptaid at designated dosage. IC_50_ is shown in relation to the IC_50_ of oxaliplatin alone.

Supplementary Table 1 Clinical data of 148 CRC patients with tissues resected for organoid preparation. Paired tissues collected from the primary and metastatic lesions of the same patients are shaded in grey.

Supplementary Table 2 Clinical data of 42 advanced CRC PDOs and their *in vitro* oxaliplatin response.

Supplementary Table 3 Clinical data of 17 CRC patients with PDOs analyzed for *in vitro* oxaliplatin response and with clinical follow-up data after FOLFOX treatment.

Supplementary Table 4 Drug response signatures identified by LOOCV & GSEA.

Supplementary Table 5 List of genes in the G2/M and mitotic spindle hallmarks that are associated with the OR PDOs. The ones in shade are commonly identified in the two hallmarks.

Supplementary Table 6 IC_50_ values of individual compounds and in combination with oxaliplatin (Oxali) in both oxaliplatin-resistnat and -seinsitve PDOs.
