# Supplementary figures and images for: Patient-Derived Organoid Serves as a Platform for Personalized Chemotherapy in Advanced Colorectal Cancer Patients
Source: Front Oncol. 2022 Jun 1;12:883437. doi: 10.3389/fonc.2022.883437 (PMC9205170; doi:10.3389/fonc.2022.883437)

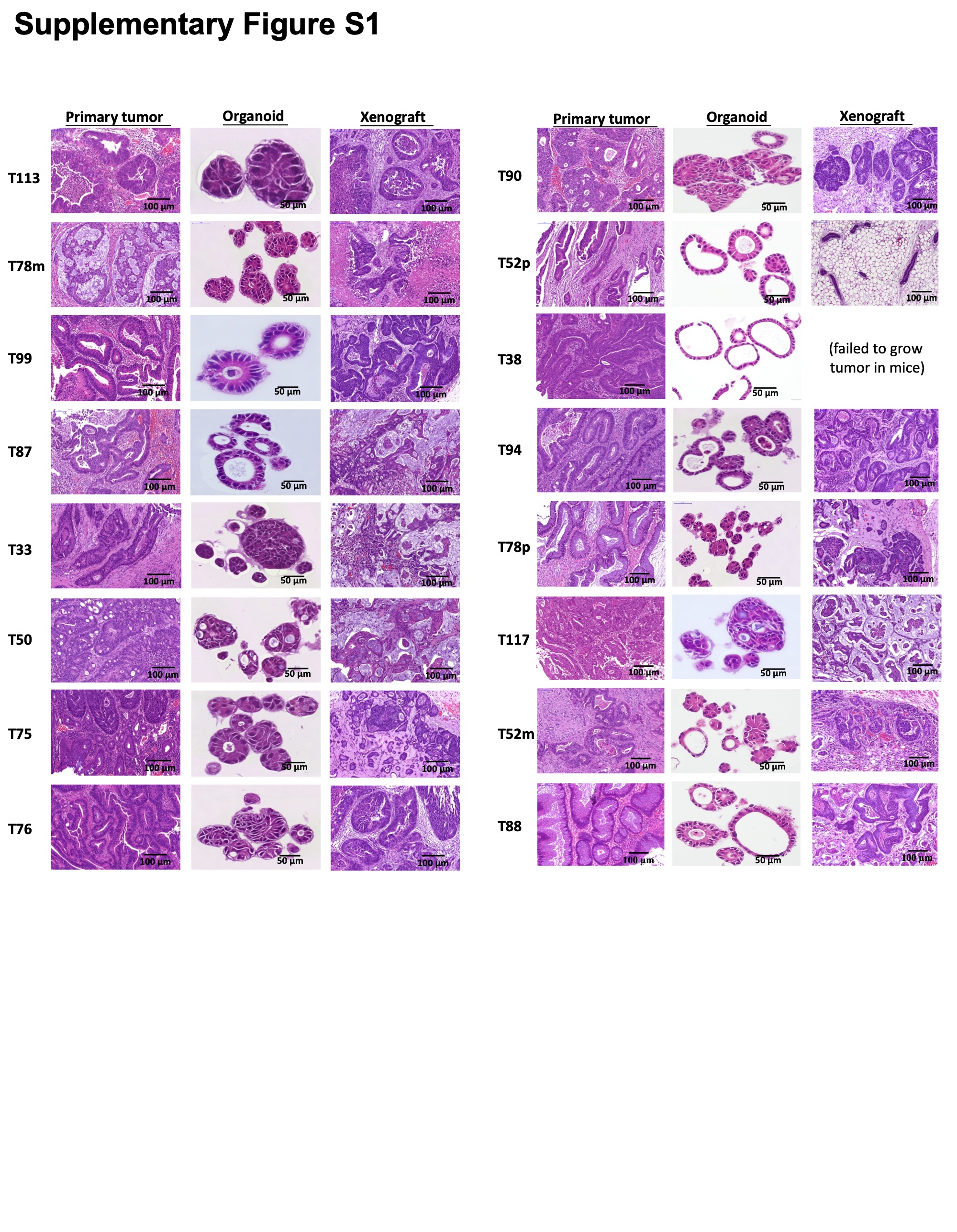

Supplement: Supplementary Figure 1 — H&E staining of primary tumors, tumor-derived organoids, and PDO xenografts in NGS mice. Scale bars: tumor tissues, 100 μm; tumor organoids, 50 μm; PDO xenograft, 100 μm. [file Image_1.jpeg]

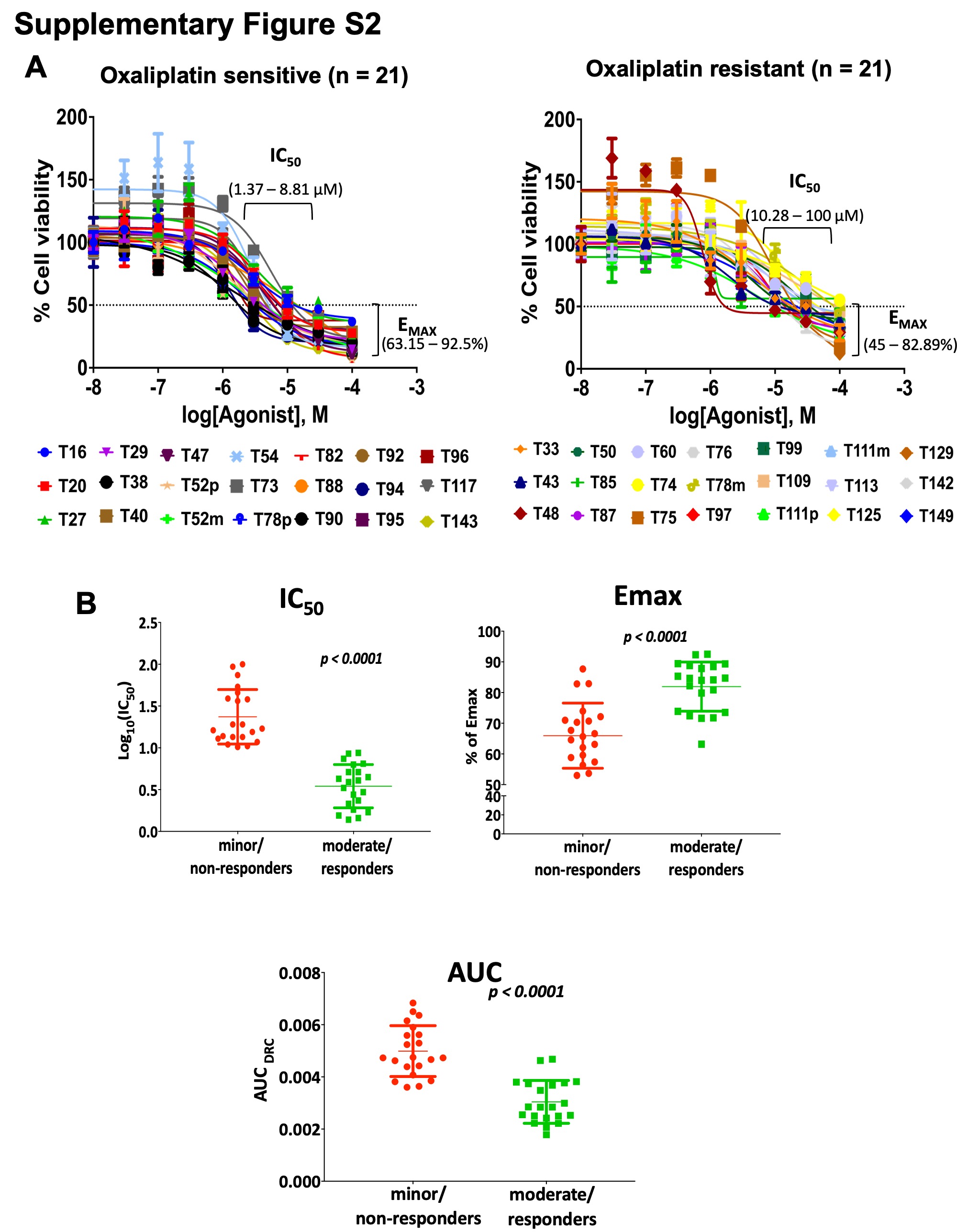

Supplement: Supplementary Figure 2 — (A) Fitted dose response curves for oxaliplatin-resistant and -sensitive PDOs. Each curve represents the mean and standard deviation of four replicates per condition. (B) Quantification of oxaliplatin response using IC50, Emax and AUC between the sensitive (strong/moderate responders) and the resistant (minor/non-responders) PDOs. Comparison was made using a two-tailed Mann-Whitney test (p < 0.0001). Dots represent individual PDOs, horizontal bars represent the mean, and error bars indicate SD. [file Image_2.jpeg]

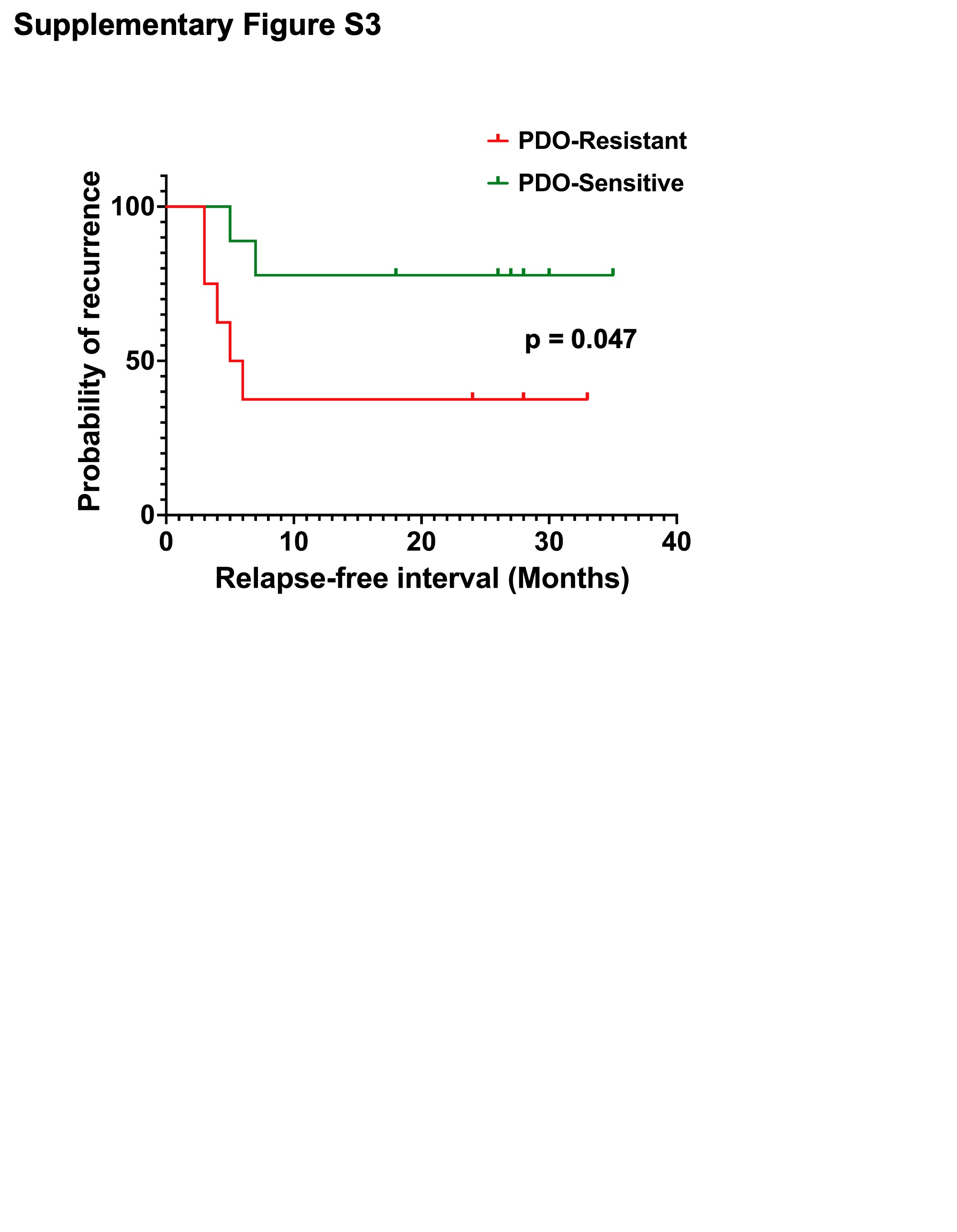

Supplement: Supplementary Figure 3 — Kaplan Meier relapse-free curve of CRC patients depending on PDO resistance or sensitivity to oxaliplatin. Comparison was made using Gehan-Breslow-Wilcoxon test (p = 0.047). This plot is based upon Fig.3B, where recurrence is given as value 1 and non-recurrence as 0. [file Image_3.jpeg]

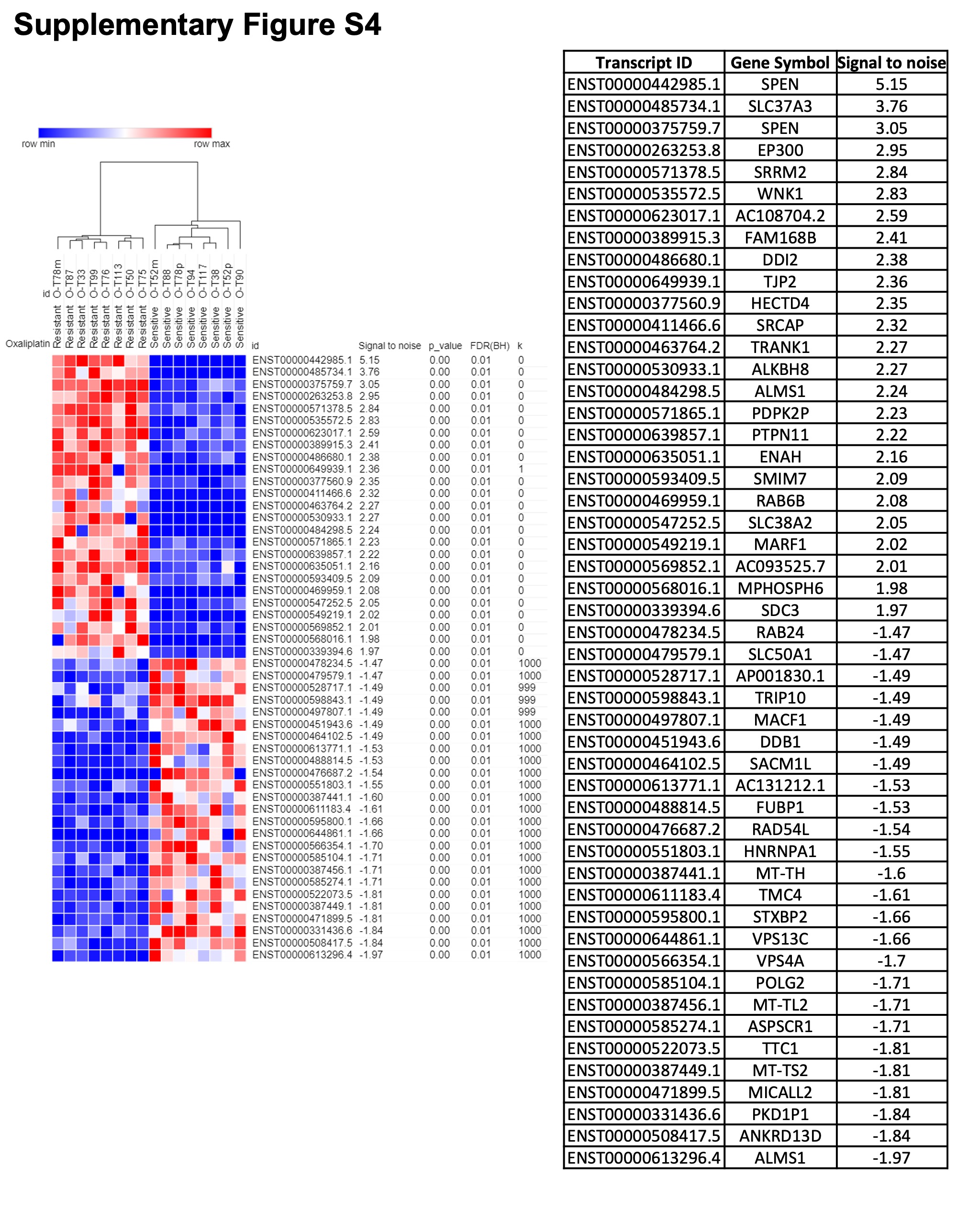

Supplement: Supplementary Figure 4 — Heatmap of top 25 up- and 25 down-regulated transcripts associated with the OR phenotype in the form of Hierarchical clustering (Spearman’s correlation) by Morpheus Marker selection method (Input: 2555 differential expressed transcripts; 1000 permutations). [file Image_4.jpeg]

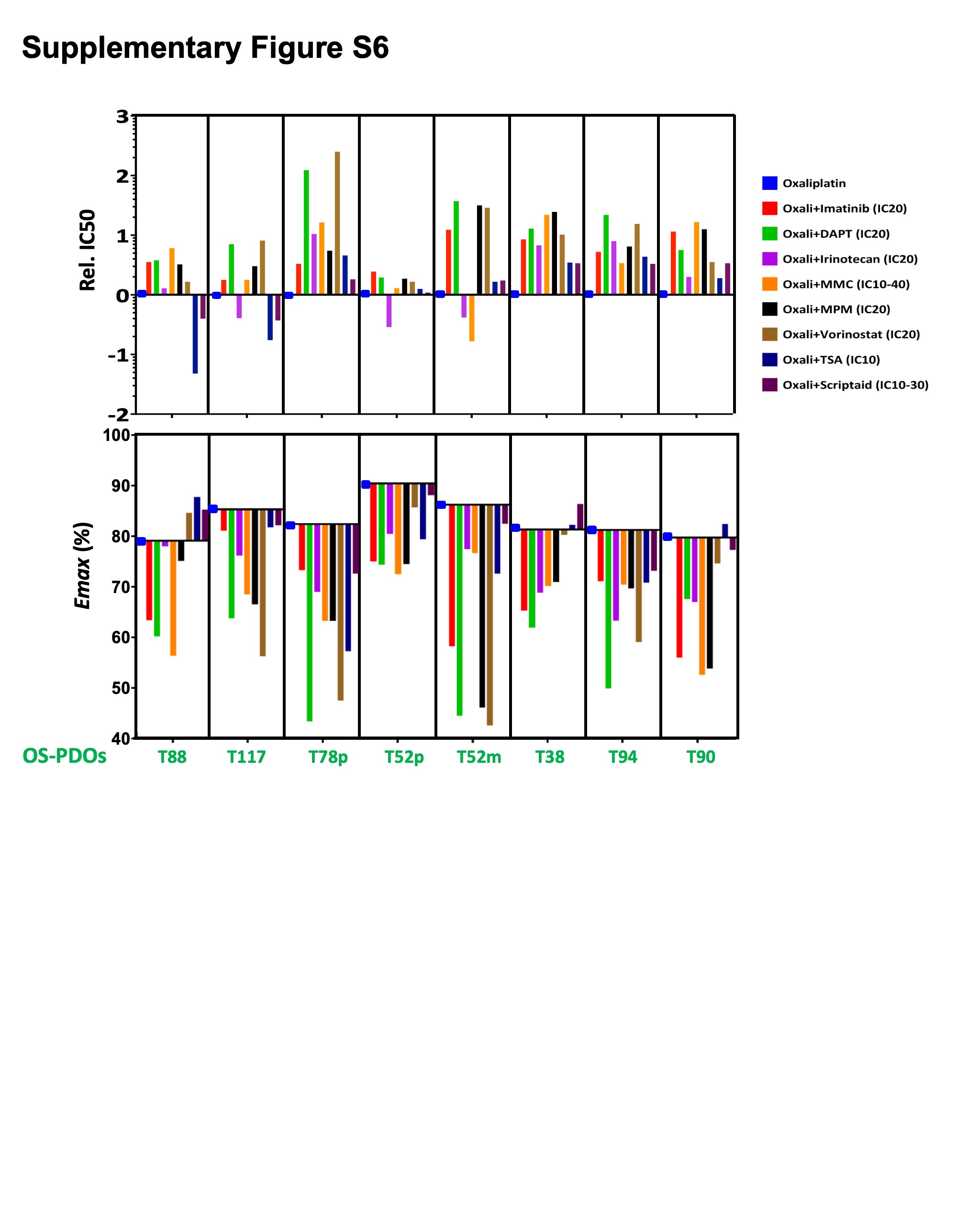

Supplement: Supplementary Figure 6 — Response to oxaliplatin-based combination therapy in OS PDOs. Bar graphs show the IC50 and Emax of each PDO towards oxaliplatin alone or in combination with imatinib, DAPT, irinotecan, mitomycin-C, mycophenolate mofetil, vorinostat, trichostatin-A, or Scriptaid at designated dosage. IC50 is shown in relation to the IC50 of oxaliplatin alone. [file Image_6.jpeg]
