# Supplementary material for: Patient-Derived Organoid Serves as a Platform for Personalized Chemotherapy in Advanced Colorectal Cancer Patients
Source: Front Oncol. 2022 Jun 1;12:883437. doi: 10.3389/fonc.2022.883437 (PMC9205170; doi:10.3389/fonc.2022.883437)
Supplement: Supplementary Figure 5 — Complete list of significant hallmarks and pathways that are upregulated in oxaliplatin-resistant and -sensitive TCGA CRC patients and CRC-PDOs. [file Presentation_5.zip › Supplementary Figure S5.pptx]

## Slide 1
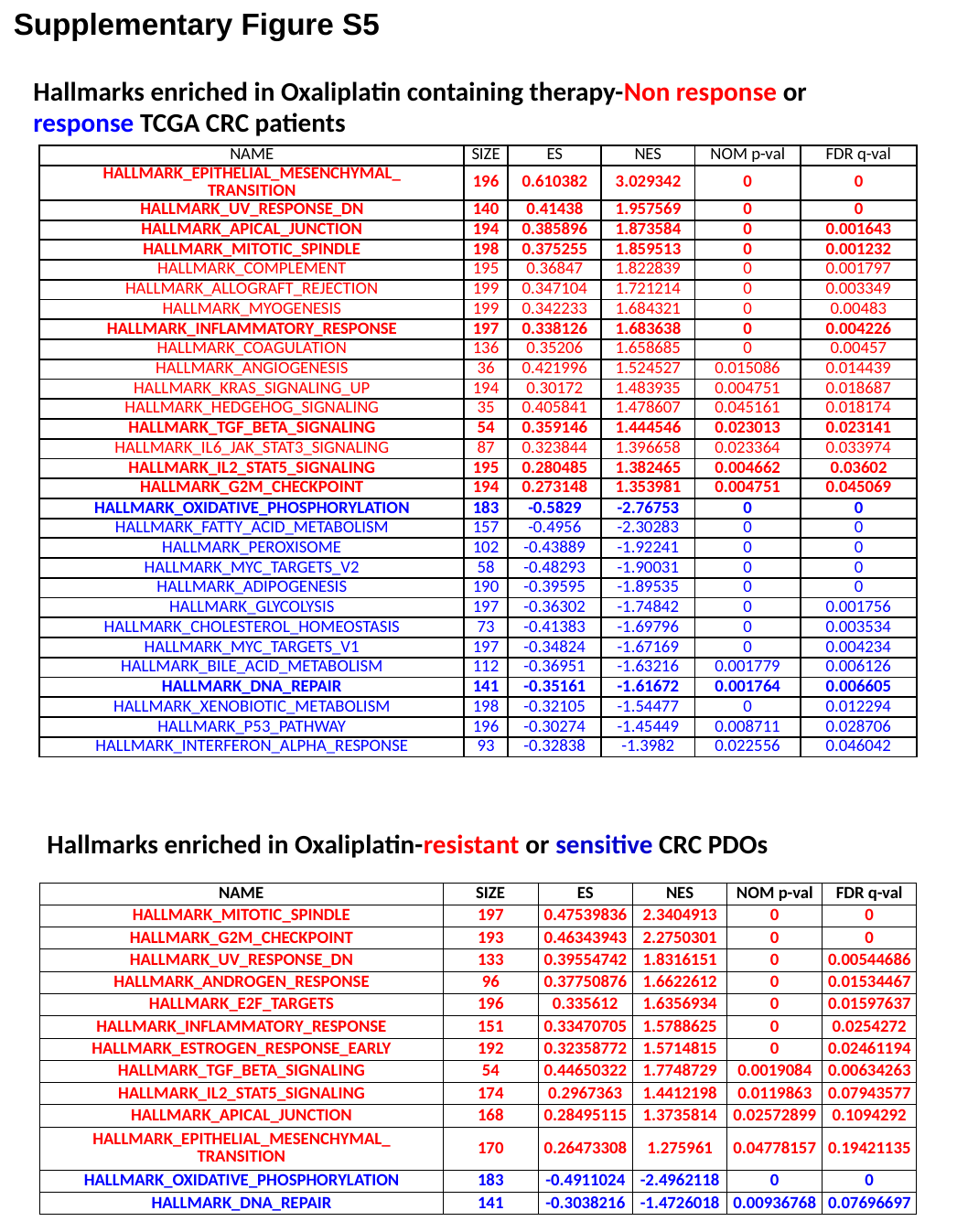

Supplementary Figure S5
Hallmarks enriched in Oxaliplatin containing therapy-Non response or response TCGA CRC patients
| NAME | SIZE | ES | NES | NOM p-val | FDR q-val |
| --- | --- | --- | --- | --- | --- |
| HALLMARK\_EPITHELIAL\_MESENCHYMAL\_ TRANSITION | 196 | 0.610382 | 3.029342 | 0 | 0 |
| HALLMARK\_UV\_RESPONSE\_DN | 140 | 0.41438 | 1.957569 | 0 | 0 |
| HALLMARK\_APICAL\_JUNCTION | 194 | 0.385896 | 1.873584 | 0 | 0.001643 |
| HALLMARK\_MITOTIC\_SPINDLE | 198 | 0.375255 | 1.859513 | 0 | 0.001232 |
| HALLMARK\_COMPLEMENT | 195 | 0.36847 | 1.822839 | 0 | 0.001797 |
| HALLMARK\_ALLOGRAFT\_REJECTION | 199 | 0.347104 | 1.721214 | 0 | 0.003349 |
| HALLMARK\_MYOGENESIS | 199 | 0.342233 | 1.684321 | 0 | 0.00483 |
| HALLMARK\_INFLAMMATORY\_RESPONSE | 197 | 0.338126 | 1.683638 | 0 | 0.004226 |
| HALLMARK\_COAGULATION | 136 | 0.35206 | 1.658685 | 0 | 0.00457 |
| HALLMARK\_ANGIOGENESIS | 36 | 0.421996 | 1.524527 | 0.015086 | 0.014439 |
| HALLMARK\_KRAS\_SIGNALING\_UP | 194 | 0.30172 | 1.483935 | 0.004751 | 0.018687 |
| HALLMARK\_HEDGEHOG\_SIGNALING | 35 | 0.405841 | 1.478607 | 0.045161 | 0.018174 |
| HALLMARK\_TGF\_BETA\_SIGNALING | 54 | 0.359146 | 1.444546 | 0.023013 | 0.023141 |
| HALLMARK\_IL6\_JAK\_STAT3\_SIGNALING | 87 | 0.323844 | 1.396658 | 0.023364 | 0.033974 |
| HALLMARK\_IL2\_STAT5\_SIGNALING | 195 | 0.280485 | 1.382465 | 0.004662 | 0.03602 |
| HALLMARK\_G2M\_CHECKPOINT | 194 | 0.273148 | 1.353981 | 0.004751 | 0.045069 |
| HALLMARK\_OXIDATIVE\_PHOSPHORYLATION | 183 | -0.5829 | -2.76753 | 0 | 0 |
| HALLMARK\_FATTY\_ACID\_METABOLISM | 157 | -0.4956 | -2.30283 | 0 | 0 |
| HALLMARK\_PEROXISOME | 102 | -0.43889 | -1.92241 | 0 | 0 |
| HALLMARK\_MYC\_TARGETS\_V2 | 58 | -0.48293 | -1.90031 | 0 | 0 |
| HALLMARK\_ADIPOGENESIS | 190 | -0.39595 | -1.89535 | 0 | 0 |
| HALLMARK\_GLYCOLYSIS | 197 | -0.36302 | -1.74842 | 0 | 0.001756 |
| HALLMARK\_CHOLESTEROL\_HOMEOSTASIS | 73 | -0.41383 | -1.69796 | 0 | 0.003534 |
| HALLMARK\_MYC\_TARGETS\_V1 | 197 | -0.34824 | -1.67169 | 0 | 0.004234 |
| HALLMARK\_BILE\_ACID\_METABOLISM | 112 | -0.36951 | -1.63216 | 0.001779 | 0.006126 |
| HALLMARK\_DNA\_REPAIR | 141 | -0.35161 | -1.61672 | 0.001764 | 0.006605 |
| HALLMARK\_XENOBIOTIC\_METABOLISM | 198 | -0.32105 | -1.54477 | 0 | 0.012294 |
| HALLMARK\_P53\_PATHWAY | 196 | -0.30274 | -1.45449 | 0.008711 | 0.028706 |
| HALLMARK\_INTERFERON\_ALPHA\_RESPONSE | 93 | -0.32838 | -1.3982 | 0.022556 | 0.046042 |
Hallmarks enriched in Oxaliplatin-resistant or sensitive CRC PDOs
| NAME | SIZE | ES | NES | NOM p-val | FDR q-val |
| --- | --- | --- | --- | --- | --- |
| HALLMARK\_MITOTIC\_SPINDLE | 197 | 0.47539836 | 2.3404913 | 0 | 0 |
| HALLMARK\_G2M\_CHECKPOINT | 193 | 0.46343943 | 2.2750301 | 0 | 0 |
| HALLMARK\_UV\_RESPONSE\_DN | 133 | 0.39554742 | 1.8316151 | 0 | 0.00544686 |
| HALLMARK\_ANDROGEN\_RESPONSE | 96 | 0.37750876 | 1.6622612 | 0 | 0.01534467 |
| HALLMARK\_E2F\_TARGETS | 196 | 0.335612 | 1.6356934 | 0 | 0.01597637 |
| HALLMARK\_INFLAMMATORY\_RESPONSE | 151 | 0.33470705 | 1.5788625 | 0 | 0.0254272 |
| HALLMARK\_ESTROGEN\_RESPONSE\_EARLY | 192 | 0.32358772 | 1.5714815 | 0 | 0.02461194 |
| HALLMARK\_TGF\_BETA\_SIGNALING | 54 | 0.44650322 | 1.7748729 | 0.0019084 | 0.00634263 |
| HALLMARK\_IL2\_STAT5\_SIGNALING | 174 | 0.2967363 | 1.4412198 | 0.0119863 | 0.07943577 |
| HALLMARK\_APICAL\_JUNCTION | 168 | 0.28495115 | 1.3735814 | 0.02572899 | 0.1094292 |
| HALLMARK\_EPITHELIAL\_MESENCHYMAL\_ TRANSITION | 170 | 0.26473308 | 1.275961 | 0.04778157 | 0.19421135 |
| HALLMARK\_OXIDATIVE\_PHOSPHORYLATION | 183 | -0.4911024 | -2.4962118 | 0 | 0 |
| HALLMARK\_DNA\_REPAIR | 141 | -0.3038216 | -1.4726018 | 0.00936768 | 0.07696697 |

## Slide 2
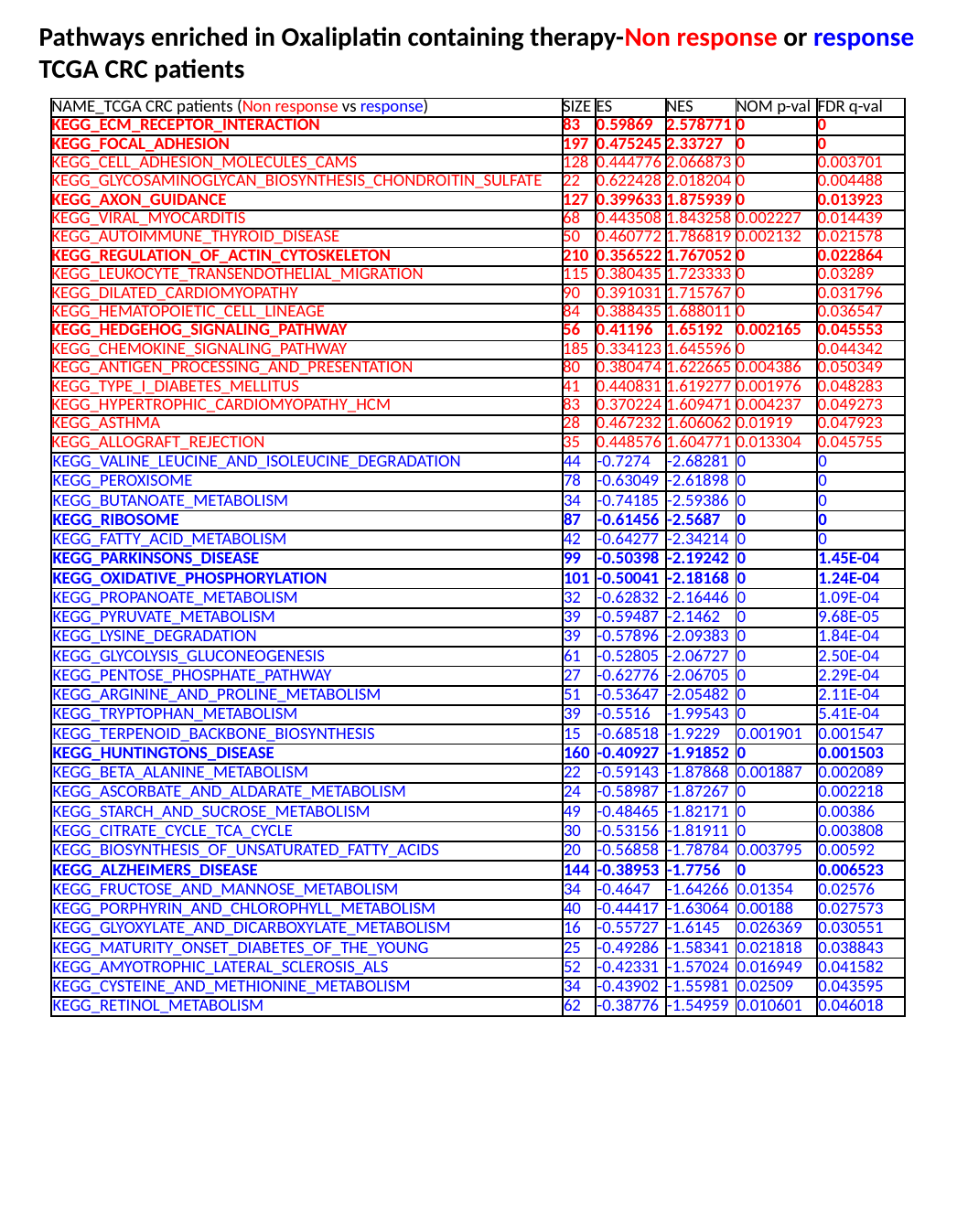

Pathways enriched in Oxaliplatin containing therapy-Non response or response TCGA CRC patients
| NAME\_TCGA CRC patients (Non response vs response) | SIZE | ES | NES | NOM p-val | FDR q-val |
| --- | --- | --- | --- | --- | --- |
| KEGG\_ECM\_RECEPTOR\_INTERACTION | 83 | 0.59869 | 2.578771 | 0 | 0 |
| KEGG\_FOCAL\_ADHESION | 197 | 0.475245 | 2.33727 | 0 | 0 |
| KEGG\_CELL\_ADHESION\_MOLECULES\_CAMS | 128 | 0.444776 | 2.066873 | 0 | 0.003701 |
| KEGG\_GLYCOSAMINOGLYCAN\_BIOSYNTHESIS\_CHONDROITIN\_SULFATE | 22 | 0.622428 | 2.018204 | 0 | 0.004488 |
| KEGG\_AXON\_GUIDANCE | 127 | 0.399633 | 1.875939 | 0 | 0.013923 |
| KEGG\_VIRAL\_MYOCARDITIS | 68 | 0.443508 | 1.843258 | 0.002227 | 0.014439 |
| KEGG\_AUTOIMMUNE\_THYROID\_DISEASE | 50 | 0.460772 | 1.786819 | 0.002132 | 0.021578 |
| KEGG\_REGULATION\_OF\_ACTIN\_CYTOSKELETON | 210 | 0.356522 | 1.767052 | 0 | 0.022864 |
| KEGG\_LEUKOCYTE\_TRANSENDOTHELIAL\_MIGRATION | 115 | 0.380435 | 1.723333 | 0 | 0.03289 |
| KEGG\_DILATED\_CARDIOMYOPATHY | 90 | 0.391031 | 1.715767 | 0 | 0.031796 |
| KEGG\_HEMATOPOIETIC\_CELL\_LINEAGE | 84 | 0.388435 | 1.688011 | 0 | 0.036547 |
| KEGG\_HEDGEHOG\_SIGNALING\_PATHWAY | 56 | 0.41196 | 1.65192 | 0.002165 | 0.045553 |
| KEGG\_CHEMOKINE\_SIGNALING\_PATHWAY | 185 | 0.334123 | 1.645596 | 0 | 0.044342 |
| KEGG\_ANTIGEN\_PROCESSING\_AND\_PRESENTATION | 80 | 0.380474 | 1.622665 | 0.004386 | 0.050349 |
| KEGG\_TYPE\_I\_DIABETES\_MELLITUS | 41 | 0.440831 | 1.619277 | 0.001976 | 0.048283 |
| KEGG\_HYPERTROPHIC\_CARDIOMYOPATHY\_HCM | 83 | 0.370224 | 1.609471 | 0.004237 | 0.049273 |
| KEGG\_ASTHMA | 28 | 0.467232 | 1.606062 | 0.01919 | 0.047923 |
| KEGG\_ALLOGRAFT\_REJECTION | 35 | 0.448576 | 1.604771 | 0.013304 | 0.045755 |
| KEGG\_VALINE\_LEUCINE\_AND\_ISOLEUCINE\_DEGRADATION | 44 | -0.7274 | -2.68281 | 0 | 0 |
| KEGG\_PEROXISOME | 78 | -0.63049 | -2.61898 | 0 | 0 |
| KEGG\_BUTANOATE\_METABOLISM | 34 | -0.74185 | -2.59386 | 0 | 0 |
| KEGG\_RIBOSOME | 87 | -0.61456 | -2.5687 | 0 | 0 |
| KEGG\_FATTY\_ACID\_METABOLISM | 42 | -0.64277 | -2.34214 | 0 | 0 |
| KEGG\_PARKINSONS\_DISEASE | 99 | -0.50398 | -2.19242 | 0 | 1.45E-04 |
| KEGG\_OXIDATIVE\_PHOSPHORYLATION | 101 | -0.50041 | -2.18168 | 0 | 1.24E-04 |
| KEGG\_PROPANOATE\_METABOLISM | 32 | -0.62832 | -2.16446 | 0 | 1.09E-04 |
| KEGG\_PYRUVATE\_METABOLISM | 39 | -0.59487 | -2.1462 | 0 | 9.68E-05 |
| KEGG\_LYSINE\_DEGRADATION | 39 | -0.57896 | -2.09383 | 0 | 1.84E-04 |
| KEGG\_GLYCOLYSIS\_GLUCONEOGENESIS | 61 | -0.52805 | -2.06727 | 0 | 2.50E-04 |
| KEGG\_PENTOSE\_PHOSPHATE\_PATHWAY | 27 | -0.62776 | -2.06705 | 0 | 2.29E-04 |
| KEGG\_ARGININE\_AND\_PROLINE\_METABOLISM | 51 | -0.53647 | -2.05482 | 0 | 2.11E-04 |
| KEGG\_TRYPTOPHAN\_METABOLISM | 39 | -0.5516 | -1.99543 | 0 | 5.41E-04 |
| KEGG\_TERPENOID\_BACKBONE\_BIOSYNTHESIS | 15 | -0.68518 | -1.9229 | 0.001901 | 0.001547 |
| KEGG\_HUNTINGTONS\_DISEASE | 160 | -0.40927 | -1.91852 | 0 | 0.001503 |
| KEGG\_BETA\_ALANINE\_METABOLISM | 22 | -0.59143 | -1.87868 | 0.001887 | 0.002089 |
| KEGG\_ASCORBATE\_AND\_ALDARATE\_METABOLISM | 24 | -0.58987 | -1.87267 | 0 | 0.002218 |
| KEGG\_STARCH\_AND\_SUCROSE\_METABOLISM | 49 | -0.48465 | -1.82171 | 0 | 0.00386 |
| KEGG\_CITRATE\_CYCLE\_TCA\_CYCLE | 30 | -0.53156 | -1.81911 | 0 | 0.003808 |
| KEGG\_BIOSYNTHESIS\_OF\_UNSATURATED\_FATTY\_ACIDS | 20 | -0.56858 | -1.78784 | 0.003795 | 0.00592 |
| KEGG\_ALZHEIMERS\_DISEASE | 144 | -0.38953 | -1.7756 | 0 | 0.006523 |
| KEGG\_FRUCTOSE\_AND\_MANNOSE\_METABOLISM | 34 | -0.4647 | -1.64266 | 0.01354 | 0.02576 |
| KEGG\_PORPHYRIN\_AND\_CHLOROPHYLL\_METABOLISM | 40 | -0.44417 | -1.63064 | 0.00188 | 0.027573 |
| KEGG\_GLYOXYLATE\_AND\_DICARBOXYLATE\_METABOLISM | 16 | -0.55727 | -1.6145 | 0.026369 | 0.030551 |
| KEGG\_MATURITY\_ONSET\_DIABETES\_OF\_THE\_YOUNG | 25 | -0.49286 | -1.58341 | 0.021818 | 0.038843 |
| KEGG\_AMYOTROPHIC\_LATERAL\_SCLEROSIS\_ALS | 52 | -0.42331 | -1.57024 | 0.016949 | 0.041582 |
| KEGG\_CYSTEINE\_AND\_METHIONINE\_METABOLISM | 34 | -0.43902 | -1.55981 | 0.02509 | 0.043595 |
| KEGG\_RETINOL\_METABOLISM | 62 | -0.38776 | -1.54959 | 0.010601 | 0.046018 |

## Slide 3
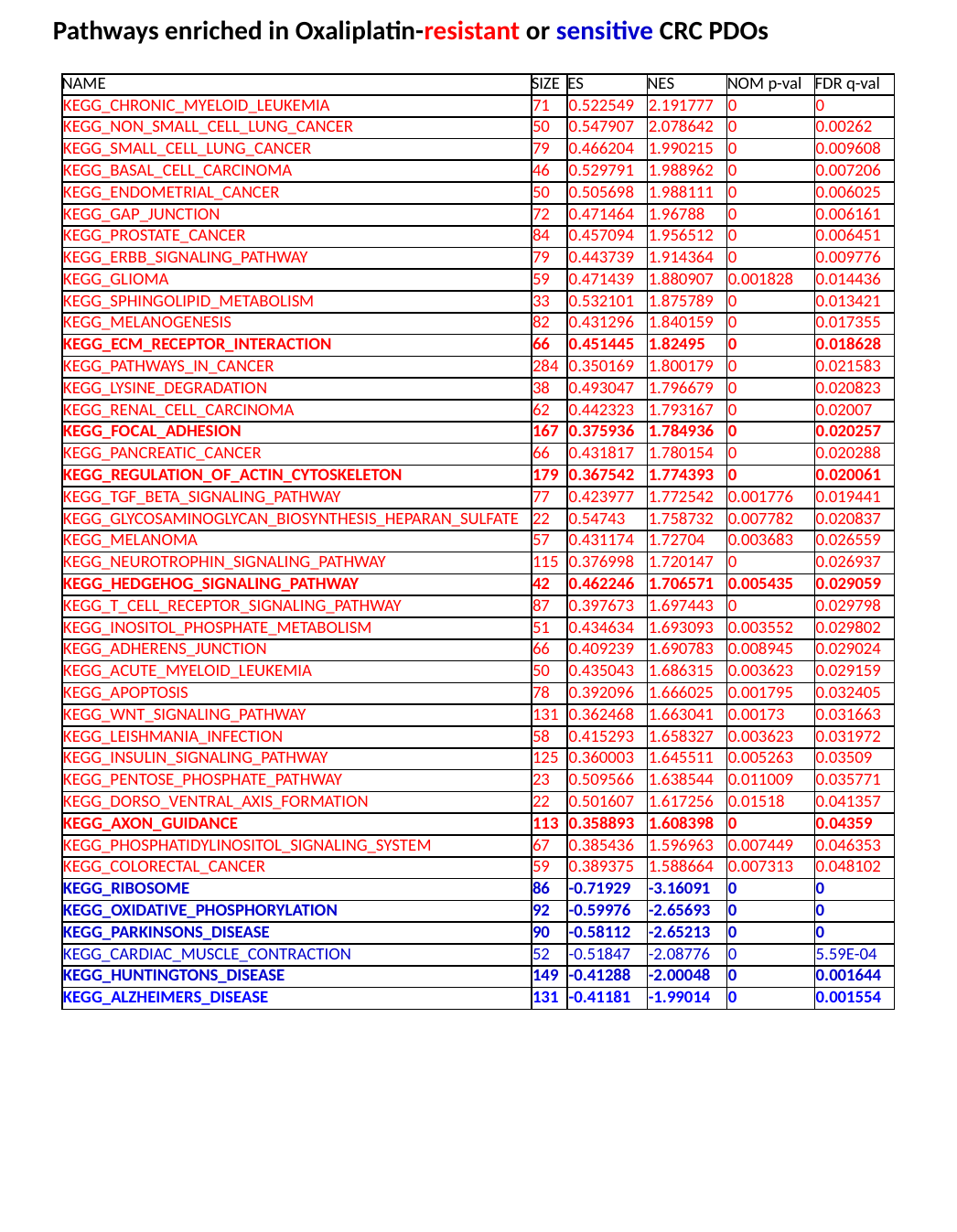

Pathways enriched in Oxaliplatin-resistant or sensitive CRC PDOs
| NAME | SIZE | ES | NES | NOM p-val | FDR q-val |
| --- | --- | --- | --- | --- | --- |
| KEGG\_CHRONIC\_MYELOID\_LEUKEMIA | 71 | 0.522549 | 2.191777 | 0 | 0 |
| KEGG\_NON\_SMALL\_CELL\_LUNG\_CANCER | 50 | 0.547907 | 2.078642 | 0 | 0.00262 |
| KEGG\_SMALL\_CELL\_LUNG\_CANCER | 79 | 0.466204 | 1.990215 | 0 | 0.009608 |
| KEGG\_BASAL\_CELL\_CARCINOMA | 46 | 0.529791 | 1.988962 | 0 | 0.007206 |
| KEGG\_ENDOMETRIAL\_CANCER | 50 | 0.505698 | 1.988111 | 0 | 0.006025 |
| KEGG\_GAP\_JUNCTION | 72 | 0.471464 | 1.96788 | 0 | 0.006161 |
| KEGG\_PROSTATE\_CANCER | 84 | 0.457094 | 1.956512 | 0 | 0.006451 |
| KEGG\_ERBB\_SIGNALING\_PATHWAY | 79 | 0.443739 | 1.914364 | 0 | 0.009776 |
| KEGG\_GLIOMA | 59 | 0.471439 | 1.880907 | 0.001828 | 0.014436 |
| KEGG\_SPHINGOLIPID\_METABOLISM | 33 | 0.532101 | 1.875789 | 0 | 0.013421 |
| KEGG\_MELANOGENESIS | 82 | 0.431296 | 1.840159 | 0 | 0.017355 |
| KEGG\_ECM\_RECEPTOR\_INTERACTION | 66 | 0.451445 | 1.82495 | 0 | 0.018628 |
| KEGG\_PATHWAYS\_IN\_CANCER | 284 | 0.350169 | 1.800179 | 0 | 0.021583 |
| KEGG\_LYSINE\_DEGRADATION | 38 | 0.493047 | 1.796679 | 0 | 0.020823 |
| KEGG\_RENAL\_CELL\_CARCINOMA | 62 | 0.442323 | 1.793167 | 0 | 0.02007 |
| KEGG\_FOCAL\_ADHESION | 167 | 0.375936 | 1.784936 | 0 | 0.020257 |
| KEGG\_PANCREATIC\_CANCER | 66 | 0.431817 | 1.780154 | 0 | 0.020288 |
| KEGG\_REGULATION\_OF\_ACTIN\_CYTOSKELETON | 179 | 0.367542 | 1.774393 | 0 | 0.020061 |
| KEGG\_TGF\_BETA\_SIGNALING\_PATHWAY | 77 | 0.423977 | 1.772542 | 0.001776 | 0.019441 |
| KEGG\_GLYCOSAMINOGLYCAN\_BIOSYNTHESIS\_HEPARAN\_SULFATE | 22 | 0.54743 | 1.758732 | 0.007782 | 0.020837 |
| KEGG\_MELANOMA | 57 | 0.431174 | 1.72704 | 0.003683 | 0.026559 |
| KEGG\_NEUROTROPHIN\_SIGNALING\_PATHWAY | 115 | 0.376998 | 1.720147 | 0 | 0.026937 |
| KEGG\_HEDGEHOG\_SIGNALING\_PATHWAY | 42 | 0.462246 | 1.706571 | 0.005435 | 0.029059 |
| KEGG\_T\_CELL\_RECEPTOR\_SIGNALING\_PATHWAY | 87 | 0.397673 | 1.697443 | 0 | 0.029798 |
| KEGG\_INOSITOL\_PHOSPHATE\_METABOLISM | 51 | 0.434634 | 1.693093 | 0.003552 | 0.029802 |
| KEGG\_ADHERENS\_JUNCTION | 66 | 0.409239 | 1.690783 | 0.008945 | 0.029024 |
| KEGG\_ACUTE\_MYELOID\_LEUKEMIA | 50 | 0.435043 | 1.686315 | 0.003623 | 0.029159 |
| KEGG\_APOPTOSIS | 78 | 0.392096 | 1.666025 | 0.001795 | 0.032405 |
| KEGG\_WNT\_SIGNALING\_PATHWAY | 131 | 0.362468 | 1.663041 | 0.00173 | 0.031663 |
| KEGG\_LEISHMANIA\_INFECTION | 58 | 0.415293 | 1.658327 | 0.003623 | 0.031972 |
| KEGG\_INSULIN\_SIGNALING\_PATHWAY | 125 | 0.360003 | 1.645511 | 0.005263 | 0.03509 |
| KEGG\_PENTOSE\_PHOSPHATE\_PATHWAY | 23 | 0.509566 | 1.638544 | 0.011009 | 0.035771 |
| KEGG\_DORSO\_VENTRAL\_AXIS\_FORMATION | 22 | 0.501607 | 1.617256 | 0.01518 | 0.041357 |
| KEGG\_AXON\_GUIDANCE | 113 | 0.358893 | 1.608398 | 0 | 0.04359 |
| KEGG\_PHOSPHATIDYLINOSITOL\_SIGNALING\_SYSTEM | 67 | 0.385436 | 1.596963 | 0.007449 | 0.046353 |
| KEGG\_COLORECTAL\_CANCER | 59 | 0.389375 | 1.588664 | 0.007313 | 0.048102 |
| KEGG\_RIBOSOME | 86 | -0.71929 | -3.16091 | 0 | 0 |
| KEGG\_OXIDATIVE\_PHOSPHORYLATION | 92 | -0.59976 | -2.65693 | 0 | 0 |
| KEGG\_PARKINSONS\_DISEASE | 90 | -0.58112 | -2.65213 | 0 | 0 |
| KEGG\_CARDIAC\_MUSCLE\_CONTRACTION | 52 | -0.51847 | -2.08776 | 0 | 5.59E-04 |
| KEGG\_HUNTINGTONS\_DISEASE | 149 | -0.41288 | -2.00048 | 0 | 0.001644 |
| KEGG\_ALZHEIMERS\_DISEASE | 131 | -0.41181 | -1.99014 | 0 | 0.001554 |
