# Supplementary material for: Patient-Derived Organoid Serves as a Platform for Personalized Chemotherapy in Advanced Colorectal Cancer Patients
Source: Front Oncol. 2022 Jun 1;12:883437. doi: 10.3389/fonc.2022.883437 (PMC9205170; doi:10.3389/fonc.2022.883437)
Supplement: Supplementary Table 1 — Clinical data of 148 CRC patients with tissues resected for organoid preparation. Paired tissues collected from the primary and metastatic lesions of the same patients are shaded in grey. [file Table_1.docx]

**Supplementary Table S1**. Clinical data of patients involved in study

| **Sample ID** | **Gender** | **Age** | **Stage (TNM)** | **Sample site** |
| --- | --- | --- | --- | --- |
| CRC-0001 | M | 72 | stage IIIA (T3 N2 M0) | Rectum cancer |
| CRC-0002 | M | 67 | stage I (T2 N0 M0) | Hepatic flexure adenoma with high grade dysplasia |
| CRC-0003 | F | 62 | stage III (T4a N2 M0) | Colon cancer |
| CRC-0004 | F | 56 | stage I (T2 N0 M0) | Sigmoid colon cancer |
| CRC-0005 | F | 69 | stage IVB (T2 N0 M1b) | Sigmoid colon cancer with meta', obstruction s/p T-loop colostomy |
| CRC-0006 | M | 49 | stage IIIA (T2 N1 M0) | Rectal cancer |
| CRC-0007 | F | 78 | stage I (T2 N0 M0) | Synchronous colon cancer at ascending colon and rectum |
| CRC-0008 | F | 72 | stage I (T2 N0 M0) | Sigmoid colon cancer |
| CRC-0009 | M | 67 | stage IIIB (T3 N2a M0) | Sigmoid colon cancer |
| CRC-0010 | M | 64 | stage IIIA (T2 N1 M0) | Rectal cancer |
| CRC-0011 | M | 75 | stage IIA (T3 N0 M0) | Rectal cancer |
| CRC-0012 | M | 63 | stage IIA (T3 N0 M0) | Rectal cancer |
| CRC-0013 | M | 75 | stage IIIB (T3 N1 M0) | Colon cancer |
| CRC-0014 | F | 36 | stage IIIB (T2 N2a M0) | Recto-Sigmoid colon cancer |
| CRC-0015 | M | 57 | stage IIIC (T4a N2a M0) | Rectal cancer |
| CRC-0016 | M | 86 | stage IIIB (T3 N1a M0) | Hepatic flexure colon cancer |
| CRC-0017 | F | 32 | stage I (T1 N0 M0) | Rectum cancer |
| CRC-0018 | M | 69 | stage I (T2 N0 M0) | Transverse colon cancer |
| CRC-0019 | F | 48 | stage IIIB ( T3 N1b M0) | Colon cancer |
| CRC-0020 | F | 60 | stage IIIB (T3 N1 M0) | Colon cancer |
| CRC-0021 | F | 77 | stage IIA (T3 N0 M0) | Colon cancer |
| CRC-0022 | M | 68 | stage IIA (T3 N0 M0) | Colon cancer |
| CRC-0023 | F | 82 | stage IIIC (T4a N0 Mx) | Synchronous ascending colon and rectal-sigmoid junction tumor (AR+RH) |
| CRC-0024 | M | 54 | stage I (T1 N0 Mx) | Rectum cancer |
| CRC-0025 | F | 77 | stage IIIA (T1 N1a Mx) | Ascending colon cancer |
| CRC-0026 | F | 77 | stage IIB (T4a N0 Mx) | Ascending colon cancer |
| CRC-0027 | M | 46 | stage IVA (T4a N1b M1) | Sigmoid colon cancer with liver meta' |
| CRC-0028 | M | 64 | stage I (T2 N0 Mx) | Sigmoid colon cancer |
| CRC-0029 | M | 76 | stage IIIA (T3 N2a Mx) | Descending-Sigmoid colon cancer |
| CRC-0030 | F | 63 | stage I (T2 N0 Mx) | Rectal-Sigmoid colon cancer |
| CRC-0031 | M | 66 | stage 0 (Tis N0 Mx) | Benign neoplasm of colon |
| CRC-0032 | F | 42 | stage IIIA (T1 N1a M0) | Sigmoid colon cancer |
| CRC-0033 | M | 74 | stage IIIB (T3 N1a Mx) | Ascending colon cancer |
| CRC-0034 | F | 73 | stage IIA (T3 N0 M0) | Sigmoid colon adenocarcinoma T |
| CRC-0035 | M | 67 | stage IIA (T3 N1a M0) | Colon cancer |
| CRC-0036 | M | 58 | stage IIIC (T4b N1b Mx) | Sigmoid colon cancer |
| CRC-0037 | M | 42 | stage 0 (Tis N0 Mx) | Colon cancer (Cecum + Ascending colon) |
| CRC-0038 | F | 45 | stage IIIA (T2 N1b Mx) | Descending colon cancer |
| CRC-0039 | F | 49 | stageIVA (T4a N2a M1c) | Sigmoid colon tumor |
| CRC-0040 | F | 65 | stage IIIA (T4a N1b Mx) | Colon cancer |
| CRC-0041 | F | 59 | stage IIIB (T3 N2a Mx) | Colon cancer |
| CRC-0042 | M | 78 | stage IIA (T3 N0 Mx) | Sigmoid colon cancer |
| CRC-0043 | F | 50 | stage IIIB (T3 N1b Mx) | Sigmoid colon tumor |
| CRC-0044 | F | 38 | stage IIIA (T4a N1b Mx) | Colon cancer over hepatic flexure |
| CRC-0045 | M | 65 | stage I (T2 N0 M0) | Transverse colon cancer |
| CRC-0046 | F | 58 | stage IIIB (T3 N1 M0) | Ascending colon cancer |
| CRC-0047 | F | 35 | stage IIIB (T3 N1 M0) | Sigmoid colon cancer |
| CRC-0048 | F | 83 | stage IIIB (T3 N1 M0) | Cecum cancer |
| CRC-0049 | F | 73 | stage IIB (T3 N0 M0) | Hepatic colon cancer |
| CRC-0050 | M | 71 | stage IIIC (T4a N2a Mx) | Cecum cancer |
| CRC-0051 | M | 38 | stage IIIB (T3 N2b Mx) | Transverse colon cancer |
| CRC-0052 | F | 80 | stage IVA (T3 N2a M1) | Sigmoid colon cancer with liver meta' (S2/4/4b/7/8)-Colon |
| CRC-0053 | F | 80 | stage IVA (T3 N2a M1) | Sigmoid colon cancer with liver meta' (S2/4/4b/7/8)-Liver |
| CRC-0054 | M | 41 | stage IVA (T4a N2a M1) | Sigmoid colon cancer with liver, lung meta' |
| CRC-0055 | F | 60 | stage IIB (T3 N0 Mx) | Descending colon cancer |
| CRC-0056 | F | 37 | stage IIA (T3 N0 Mx) | Colon cancer at hepatic flexure |
| CRC-0057 | M | 82 | stage IIB (T4 N0 Mx) | Sigmoid colon cancer |
| CRC-0058 | F | 58 | stage IIA (T3 N0 Mx) | Sigmoid colon cancer |
| CRC-0059 | M | 69 | stage IIB (T4 N0 Mx) | Sigmoid colon cancer |
| CRC-0060 | M | 46 | stage IVA (T4 N0 M1) | colon cancer |
| CRC-0061 | M | 71 | stage IIB (T3 N0 Mx) | Rectal cancer and sessile polyp |
| CRC-0062 | M | 75 | stage I (T2 N0 Mx) | Sigmoid colon cancer |
| CRC-0063 | M | 66 | stage IIB (T3 N0 Mx) | Rectal cancer |
| CRC-0064 | M | 65 | stage I (T2 N0 Mx) | Ascending colon tumor |
| CRC-0065 | M | 39 | stage IIB (T3 N0 Mx) | Sigmoid colon cancer |
| CRC-0066 | M | 48 | stage IIB (T3 N0 Mx) | Ascending colon cancer |
| CRC-0067 | F | 57 | stage IVA (T2 N0 M1a) | Sigmoid colon cancer with liver meta' (Primary site-colon) |
| CRC-0068 | F | 57 | stage IVA (T2 N0 M1a) | Sigmoid colon cancer with liver meta' (Metastasis site-liver) |
| CRC-0069 | M | 67 | stage I (T1 N0 Mx) | Colon cancer |
| CRC-0070 | F | 84 | stage I (T1 N0 Mx) | Colon tumor |
| CRC-0071 | F | 80 | stage I (T1 N0 Mx) | Ascending colon cancer |
| CRC-0072 | F | 80 | stage IIB (T3 N0 Mx) | Sigmoid colon cancer |
| CRC-0073 | F | 64 | stage IVB (T4a N2a M1b) | Sigmoid colon tumor |
| CRC-0074 | M | 66 | stage IVA (T4a N2a M1c) | Colon cancer |
| CRC-0075 | M | 65 | stage IIIB (T3 N1b Mx) | Colon cancer |
| CRC-0076 | F | 78 | stage IVA (T4a N2a M1c) | Sigmoid colon cancer |
| CRC-0077 | F | 62 | stage IVA (T3 N2a M1) | Descending colon cancer with liver metastasis (Metastasis site-liver) |
| CRC-0078 | F | 62 | stage IVA (T3 N2a M1) | Descending colon cancer with liver metastasis (Primary site-colon) |
| CRC-0079 | M | 81 | stage I (T2 N0 Mx) | Cecum and hepatic flexure colon cancer |
| CRC-0080 | F | 67 | stage IVA (T4a N2a M1c) | Cecal cancer with tumor seeding over rectum |
| CRC-0081 | M | 34 | stage IIB (T3 N0 Mx) | Descending colon cancer |
| CRC-0082 | M | 69 | stage IIIA (T2 N1 M0) | Recto-Sigmoid junction colon cancer |
| CRC-0083 | M | 48 | stage I (T1 N0 Mx) | Sigmoid colon cancer |
| CRC-0084 | M | 58 | stage IIIA (T3 N2 M0) | Ascending colon cancer |
| CRC-0085 | F | 49 | stage IIIB (T3 N1b Mx) | Rectum cancer |
| CRC-0086 | F | 67 | stage IIB (T3 N0 Mx) | Rectal cancer |
| CRC-0087 | M | 87 | stage IIIA (T3 N2 M0) | Colon cancer |
| CRC-0088 | F | 60 | stage IIIA (T3 N2 M0) | Sigmoid colon cancer |
| CRC-0089 | F | 46 | stage IIIA (T3 N2 M0) | Cecum cancer |
| CRC-0090 | M | 77 | stage IIIA (T3 N2 M0) | Cecum cancer |
| CRC-0091 | F | 72 | stage I (T1 N0 Mx) | Sigmoid colon early cancer |
| CRC-0092 | F | 73 | stage IIIB (T3 N1a Mx) | Hepatic flexure colon cancer |
| CRC-0093 | M | 55 | stage IIB (T3 N0 Mx) | Recto-Sigmoid junction colon cancer adenocarcinoma |
| CRC-0094 | M | 65 | stage IIIC (T4a N2a Mx) | Synchronous colon cancer, splenic flexure, sigmoid colon cancer |
| CRC-0095 | M | 56 | stage IIIB (T3 N1a Mx) | Sigmoid colon cancer |
| CRC-0096 | M | 55 | stage IIIA (T4a N1a Mx) | Sigmoid colon cancer |
| CRC-0097 | M | 45 | stage IVA (T3 N1b M1) | Rectal cancer with liver meta’ (Metastasis site-liver) |
| CRC-0098 | F | 75 | stage IIB (T4 N0 Mx) | Cecum cancer |
| CRC-0099 | M | 55 | stage IVA (T4a N1b M1) | Rectal cancer |
| CRC-0100 | M | 84 | stage IIIB (T3 N1c Mx) | Sigmoid colon cancer |
| CRC-0101 | M | 71 | stage I (T2 N0 Mx) | Sigmoid colon cancer |
| CRC-0102 | F | 66 | stage IIIB (T3 N1a Mx) | Sigmoid colon cancer |
| CRC-0103 | F | 67 | stage IIB (T3 N0 Mx) | Synchronous proximal transverse and splenic flexure colon cancer |
| CRC-0104 | M | 50 | stage I (T2 N0 Mx) | Colon cancer |
| CRC-0105 | F | 72 | stage IIB (T3 N0 Mx) | Ascending colon cancer |
| CRC-0106 | F | 79 | stage IIB (T4 N0 Mx) | Recto-Sigmoid junction colon cancer |
| CRC-0107 | F | 67 | stage IIB (T4 N0 Mx) | Sigmoid colon cancer |
| CRC-0108 | M | 67 | stage IIB (T3 N0 Mx) | Ascending colon cancer |
| CRC-0109 | M | 40 | stage IVA (T4a N1b M1) | Rectum cancer |
| CRC-0110 | F | 71 | stage IIB (T3 N0 Mx) | Rectum cancer |
| CRC-0111 | M | 50 | stage IVA (T3 N1b M1) | Rectum cancer and liver meta' (Primary site-colon) |
| CRC-0112 | M | 50 | stage IVA (T3 N1b M1) | Rectum cancer and liver meta' (Metastasis site-liver) |
| CRC-0113 | M | 21 | stage IIIA (T3 N2 M0) | Descending colon cancer |
| CRC-0114 | M | 67 | stage IIIB (T3 N1c Mx) | Sigmoid colon cancer |
| CRC-0115 | F | 87 | stage IIB (T3 N0 Mx) | Hepatic flexure colon cancer |
| CRC-0116 | M | 50 | stage IIB (T4 N0 Mx) | Ascending colon cancer |
| CRC-0117 | F | 101 | stage IIIC (T4b N1b Mx) | Descending colon cancer |
| CRC-0118 | M | 79 | stage IIIA (T3 N2 M0) | Descending colon cancer |
| CRC-0119 | M | 59 | stage I (T2 N0 Mx) | Ascending colon cancer |
| CRC-0120 | M | 54 | stage IIB (T3 N0 Mx) | Sigmoid colon cancer |
| CRC-0121 | M | 61 | stage IIB (T3 N0 Mx) | Ascending colon cancer |
| CRC-0122 | F | 58 | stage IIA (T3 N0 M0) | Ascending colon cancer |
| CRC-0123 | F | 64 | stage IIIB (T3 N2 M0) | Ascending colon cancer |
| CRC-0124 | F | 64 | stage 0 (Tis N0 M0) | Colon cancer over hepatic flexure |
| CRC-0125 | M | 60 | stage IIIC (T4bN2bM0) | Rectal cancer |
| CRC-0126 | M | 81 | stage IIIB (T3 N1 M0) | Sigmoid colon cancer |
| CRC-0127 | F | 63 | stage IIIB (T3 N1 Mx) | Sigmoid colon cancer |
| CRC-0128 | M | 77 | stage IIIB (T3 N1 Mx) | Rectum cancer |
| CRC-0129 | M | 57 | stage IVB (T4b N2b M0) | Rectum cancer |
| CRC-0130 | F | 54 | stage IIB (T3 N0 Mx) | Cecum cancer |
| CRC-0131 | F | 46 | stage IIB (T3 N0 Mx) | Transverse colon cancer |
| CRC-0132 | F | 77 | stage I (T2 N0 Mx) | Cancer of rectum |
| CRC-0133 | M | 65 | stage IVA (T4a N2a M1a) | Descending colon cancer |
| CRC-0134 | M | 68 | stage IIA (T3 N0 M0) | Sigmoid colon cancer |
| CRC-0135 | M | 68 | stage IIA (T3 N0 M0) | Sigmoid colon cancer |
| CRC-0136 | M | 68 | stage I (T1 N0 M0) | Sigmoid colon cancer |
| CRC-0137 | F | 75 | stage IIIB (T3 N1b M0) | Ascending colon cancer |
| CRC-0138 | M | 53 | stage IIA (T3 N0 M0) | Ascending colon tumor |
| CRC-0139 | M | 53 | stage IIIB (T3 N1b M0) | Colon cancer |
| CRC-0140 | F | 67 | stage IIB (T4 N0 Mx) | Colon cancer, Sigmoid |
| CRC-0141 | F | 62 | stage I (T1 N0 Mx) | Proximal transverse colon cancer |
| CRC-0142 | M | 70 | stage IVB (T4a N2b M1a) | Cecal cancer with liver metastasis (Metastasis site-liver) |
| CRC-0143 | M | 56 | stage IIIB (T4 N1b M0) | Splenic flexure colon cancer |
| CRC-0144 | M | 61 | stage IIIC (T4 N2b M0) | Ascending colon cancer |
| CRC-0145 | M | 74 | stage IVA (T4aN2aMx) | Sigmoid colon adenocarcinoma |
| CRC-0146 | M | 62 | stage IIIB (T4aN1cMx) | Ascending colon cancer |
| CRC-0147 | M | 53 | stage IVA (ypT3aN2aM1) | Ascending colon cancer and liver meta' (Metastasis site-liver) |
| CRC-0148 | M | 73 | stage IVA (T4aN2aM1) | Synchronous colon cancer at proximal Transverse & Descending colon |
| CRC-0149 | F | 85 | stage IVA (T4a N2a Mx) | Metachronous Ascending colon cancer s/p AR |
| CRC-0150 | F | 64 | stage IIB (T4 N0 Mx) | Sigmoid colon cancer |
| CRC-0151 | M | 83 | stage IIB (T4 N0 Mx) | Rectum cancer |

*Paired primary and metastatic tumors derived from the same patients were shaded in grey.
