# Supplementary material for: Patient-Derived Organoid Serves as a Platform for Personalized Chemotherapy in Advanced Colorectal Cancer Patients
Source: Front Oncol. 2022 Jun 1;12:883437. doi: 10.3389/fonc.2022.883437 (PMC9205170; doi:10.3389/fonc.2022.883437)
Supplement: Supplementary Table 2 — Clinical data of 42 advanced CRC PDOs and their in vitro oxaliplatin response. [file Table_2.docx]

**Supplementary Table S2**. Clinical data of 42 advanced CRC PDOs and their *in vitro* oxaliplatin response.

| **Patient-derived organoids (n = 42)** | | | **Oxaliplatin treatment** | | |
| --- | --- | --- | --- | --- | --- |
| **Patient ID** | **Stage** | **Tumor site** | **LOG IC_50_ (μM)** | **Emax (%)** | **Response category** |
| 109# | IVA (T4a N1b M1) | Rectum | 2.00 | 52.98 | Non-responder |
| 74 | IVA (T4a N2a M1c) | Colon | 1.97 | 45.00 | Non-responder |
| 85 | IIIB (T3 N1b Mx) | Rectum | 1.87 | 56.31 | Non-responder |
| 78m* | IVA (T3N2aM1) | Liver | 1.73 | 53.66 | Non-responder |
| 113 | IIIA (T3 N2 M0) | Descending colon | 1.66 | 59.59 | Non-responder |
| 111m*# | IVA (T3 N1b M1) | Liver | 1.59 | 57.36 | Non-responder |
| 99 | IVA (T4a N1b M1) | Rectum | 1.58 | 65.66 | Non-responder |
| 125 | IIIC (T4b N2b M0) | Rectum | 1.56 | 58.80 | Non-responder |
| 60 | IVA (T4 N0 M1) | Colon | 1.28 | 64.57 | Non-responder |
| 87 | IIIA (T3 N2 M0) | Colon | 1.28 | 70.28 | Non-responder |
| 50 | IIIC (T4a N2a Mx) | Cecum | 1.23 | 62.07 | Minor responder |
| 149 | IVA (T4a N2a Mx) | Ascending colon | 1.21 | 66.62 | Minor responder |
| 33 | IIIB (T3 N1a Mx) | Ascending colon | 1.19 | 70.98 | Minor responder |
| 97# | IVA (T3 N1b M1) | Rectum (with liver meta) | 1.14 | 67.68 | Minor responder |
| 75 | IIIB (T3 N1b Mx) | Colon | 1.13 | 82.89 | Minor responder |
| 48 | IIIB (T3 N1 M0) | Cecum | 1.13 | 70.70 | Minor responder |
| 43 | IIIB (T3 N1b Mx) | Sigmoid colon | 1.110 | 63.12 | Minor responder |
| 111p*# | IVA (T3 N1b M1) | Rectum (with liver meta) | 1.07 | 73.93 | Minor responder |
| 76 | IVA (T4a N2a M1c) | Sigmoid colon | 1.04 | 82.82 | Minor responder |
| 142# | IVB (T4a N2b M1a) | Cecal (with liver meta) | 1.02 | 72.13 | Minor responder |
| 129# | IVB(T4b N2b M0) | Rectum | 1.01 | 87.66 | Minor responder |
| 16 | IIIB (T3 N1a M0) | Hepatic flexure colon | 0.94 | 63.15 | Moderate responder |
| 27 | IVA(T4a N1b M1) | Sigmoid colon (with liver meta) | 0.93 | 73.51 | Moderate responder |
| 20 | IIIB (T3 N1 M0) | Right colon | 0.87 | 72.41 | Moderate responder |
| 82 | IIIA (T2 N1 M0) | Recto-Sigmoid junction colon | 0.81 | 92.31 | Moderate responder |
| 95 | IIIB (T3 N1a Mx) | Sigmoid colon | 0.80 | 73.87 | Moderate responder |
| 47 | IIIB (T3 N1 M0) | Sigmoid colon | 0.71 | 88.77 | Moderate responder |
| 29 | IIIA (T3 N2a Mx) | Descending-Sigmoid colon | 0.71 | 85.29 | Moderate responder |
| 92 | IIIB (T3 N1a Mx) | Hepatic flexure colon | 0.65 | 89.32 | Moderate responder |
| 40 | IIIA (T4a N1b Mx) | Colon | 0.63 | 71.61 | Moderate responder |
| 73 | IVB (T4a N2a M1b) | Sigmoid colon | 0.61 | 84.63 | Moderate responder |
| 88 | IIIA (T3 N2 M0) | Sigmoid colon | 0.59 | 80.88 | Moderate responder |
| 117 | IIIC (T4b N1b Mx) | Descending colon | 0.52 | 84.12 | Strong responder |
| 54 | IVA (T4a N2a M1) | Sigmoid colon (with liver, lung meta) | 0.47 | 84.71 | Strong responder |
| 78p* | IVA (T3N2aM1) | Descending colon (with liver meta) | 0.44 | 82.20 | Strong responder |
| 143 | IIIB (T4 N1b M0) | Splenic flexure colon | 0.37 | 89.4 | Strong responder |
| 52p* | IVA (T3 N2a M1) | Sigmoid colon (with liver meta) | 0.33 | 92.47 | Strong responder |
| 96 | IIIA (T4a N1a Mx) | Sigmoid colon | 0.26 | 71.74 | Strong responder |
| 52m* | IVA (T3 N2a M1) | Liver | 0.23 | 88.45 | Strong responder |
| 38 | IIIA (T2 N1b Mx) | Descending colon | 0.19 | 79.84 | Strong responder |
| 94 | IIIC (T4a N2a Mx) | Sigmoid colon | 0.16 | 83.97 | Strong responder |
| 90 | IIIA (T3 N2 M0) | Cecum | 0.14 | 87.82 | Strong responder |

*P and m specify the PDOs derived from the primary (p) and metastatic (m) tumors of the designated patient, others were derived from primary tumors.

#patients with neoadjuvant therapy
