# Supplementary material for: Patient-Derived Organoid Serves as a Platform for Personalized Chemotherapy in Advanced Colorectal Cancer Patients
Source: Front Oncol. 2022 Jun 1;12:883437. doi: 10.3389/fonc.2022.883437 (PMC9205170; doi:10.3389/fonc.2022.883437)
Supplement: Supplementary Table 3 — Clinical data of 17 CRC patients with PDOs analyzed for in vitro oxaliplatin response and with clinical follow-up data after FOLFOX treatment. [file Table_3.docx]

**Supplementary Table S3**. Clinical data of 17 CRC patients with PDOs analyzed for *in vitro* oxaliplatin response and with clinical follow-up data after FOLFOX treatment.

| ID | TNM stage | Gender | Metastasis | PDO generation site | Neoadjuvant treatment |
| --- | --- | --- | --- | --- | --- |
| 85 | IIIB | F | no | primary Rectum cancer | no |
| 113 | IIIA | M | no | primary Descending colon cancer | no |
| 99 | IVB | M | no | primary Rectum cancer | no |
| 60 | IVA | M | no | primary colon cancer at hepatic flexure | no |
| 33 | IIIB | M | no | primary Ascending colon cancer | no |
| 75 | IIIB | M | no | primary Colon cancer at 35cm from anal verge | no |
| 43 | IIIB | F | no | primary Sigmoid colon tumor | no |
| 142 | IVB | M | yes | liver metastatic site - Cecal cancer with liver metastasis | NEO ADJUVANT - FOLFOX |
| 82 | IIIA | M | no | primary Recto-Sigmoid junction colon cancer | no |
| 95 | IIIB | M | no | primary Sigmoid colon tumor | no |
| 92 | IIIB | F | no | primary colon cancer at hepatic flexure | no |
| 27 | IVA | M | yes | primary Sigmoid colon tumor | no |
| 20 | IIIB | F | no | primary colon cancer | no |
| 143 | IIIB | M | no | primary Splenic flexure colon cancer | no |
| 96 | IIIA | M | no | primary Sigmoid colon tumor | no |
| 94 | IIIC | M | no | primary Sigmoid colon tumor | no |
| 52 | IVA | F | yes | primary Sigmoid colon tumor | no |
|  |  |  |  | primary Sigmoid colon tumor - liver metastasis site |  |
