# Supplementary material for: Patient-Derived Organoid Serves as a Platform for Personalized Chemotherapy in Advanced Colorectal Cancer Patients
Source: Front Oncol. 2022 Jun 1;12:883437. doi: 10.3389/fonc.2022.883437 (PMC9205170; doi:10.3389/fonc.2022.883437)
Supplement: Supplementary Table 4 — Drug response signatures identified by LOOCV & GSEA. [file Table_4.docx]

**Supplementary Table S4**. Drug response signatures identified by LOOCV & GSEA

| **gene_id** | **position** | **gene_name** | **GSEA Score** | **GSEA-resistant ranking** | **GSEA-sensitive ranking** | **ref_gene_id** |
| --- | --- | --- | --- | --- | --- | --- |
| MSTRG.7775 | 57962 | **AC108704.2** | 2.25012 | 2 |  | ENSG00000279500.1 |
| MSTRG.20417 | 38330 | **MIR1281** | 2.11176 | 3 |  | ENSG00000284015.1 |
| MSTRG.20417 | 38329 | **EP300** | 2.11176 | 4 |  | ENSG00000100393.12 |
| MSTRG.25148 | 44059 | **RREB1** | 2.01042 | 5 |  | ENSG00000124782.19 |
| MSTRG.426 | 53615 | **SPEN** | 2.00365 | 6 |  | ENSG00000065526.10 |
| MSTRG.2406 | 42734 | **PRRC2C** | 1.85888 | 7 |  | ENSG00000117523.16 |
| MSTRG.13889 | 30145 | **AC134407.3** | 1.67906 | 15 |  | ENSG00000279880.1 |
| MSTRG.13889 | 30144 | **BPTF** | 1.67906 | 16 |  | ENSG00000171634.17 |
| MSTRG.27669 | 47201 | **KMT2E** | 1.64788 | 17 |  | ENSG00000005483.20 |
| MSTRG.19105 | 36672 | **SOGA1** | 1.56038 | 19 |  | ENSG00000149639.14 |
| MSTRG.1761 | 34852 | **VANGL1** | 1.55784 | 21 |  | ENSG00000173218.14 |
| MSTRG.408 | 53397 | **RSC1A1** | 1.32371 | 45 |  | ENSG00000215695.1 |
| MSTRG.408 | 53396 | **DDI2** | 1.32371 | 46 |  | ENSG00000197312.11 |
| MSTRG.30480 | 50619 | **ABL1** | 1.31926 | 52 |  | ENSG00000097007.17 |
| MSTRG.18518 | 35935 | **TRIP12** | 1.10540 | 117 |  | ENSG00000153827.13 |
| MSTRG.3177 | 52157 | **ZNF692** | -1.84458 |  | 3 | ENSG00000171163.15 |
| MSTRG.24611 | 43394 | **MZB1** | -1.90254 |  | 2 | ENSG00000170476.15 |
| MSTRG.5178 | 54759 | **UBXN1** | -0.96931 |  | 111 | ENSG00000162191.13 |
