# Supplementary material for: Patient-Derived Organoid Serves as a Platform for Personalized Chemotherapy in Advanced Colorectal Cancer Patients
Source: Front Oncol. 2022 Jun 1;12:883437. doi: 10.3389/fonc.2022.883437 (PMC9205170; doi:10.3389/fonc.2022.883437)
Supplement: Supplementary Table 5 — List of genes in the G2/M and mitotic spindle hallmarks that are associated with the OR PDOs. The ones in shade are commonly identified in the two hallmarks. [file Table_5.docx]

**Supplementary Table S5.** List of genes in the G2/M and mitotic spindle hallmarks that are associated with the OR PDOs. The ones in shade are commonly identified in the two hallmarks.

| **Mitotic Spindle (n=86**) | | **Common genes (n=22)** | **G2-M checkpoint (n=82)** | |
| --- | --- | --- | --- | --- |
| ALMS1 | ARHGEF11 | **ABL1** | SFPQ | HOXC10 |
| DYNC1H1 | ROCK1 | **NOTCH2** | HMGA1 | CDC20 |
| AKAP13 | KIF3B | **RASAL2** | CASP8AP2 | HIF1A |
| SPTAN1 | ARHGEF7 | **BRCA2** | TRA2B | MCM3 |
| SOS1 | ABR | **SMC1A** | HNRNPU | KPNB1 |
| FLNA | ITSN1 | **CENPE** | MKI67 | SLC7A1 |
| LATS1 | ECT2 | **CDC27** | SYNCRIP | SRSF2 |
| SPTBN1 | TUBGCP5 | **FBXO5** | NUP98 | MCM2 |
| PCNT | MID1 | **PLK1** | ATRX | MYBL2 |
| ARHGEF12 | ARHGAP5 | **MARCKS** | PRPF4B | ARID4A |
| MYO9B | FSCN1 | **BIRC5** | MAPK14 | H2AFX |
| CLASP1 | CTTN | **KIF11** | CCNF | HUS1 |
| DST | CNTRL | **ESPL1** | SMC2 | HIRA |
| CCDC88A | FGD4 | **BUB1** | HMGB3 | PRIM2 |
| PKD2 | CDC42EP1 | **NDC80** | POLE | XPO1 |
| BCR | TBCD | **INCENP** | CDKN2C | PLK4 |
| ARHGAP10 | TIAM1 | **KIF15** | CTCF | MTF2 |
| MYH9 | DOCK4 | **KIF4A** | BARD1 | ORC5 |
| KIF1B | FGD6 | **TTK** | MEIS1 | MCM6 |
| NF1 | EZR | **KIF2C** | SLC38A1 | EXO1 |
| CDC42BPA | SASS6 | **NUMA1** | E2F2 | STIL |
| RANBP9 | PALLD | **LMNB1** | WRN | MEIS2 |
| WASL | CLIP1 |  | MCM5 | SLC12A2 |
| ARFGEF1 | TAOK2 |  | CUL1 | UPF1 |
| RAB3GAP1 | GEMIN4 |  | SMARCC1 | PRMT5 |
| MYO1E | NIN |  | TOP1 | TNPO2 |
| TRIO | TUBGCP2 |  | PDS5B | FOXN3 |
| TLK1 | RHOF |  | HNRNPD | SS18 |
| FLNB | RAPGEF6 |  | EFNA5 | TMPO |
| PDLIM5 | DLGAP5 |  | STAG1 | MYC |
| EPB41L2 | ARHGAP29 |  | E2F3 | DR1 |
| YWHAE | RALBP1 |  | RBL1 | HMGN2 |
| WASF2 | GSN |  | ODC1 | CENPA |
| RICTOR | SYNPO |  | PURA | MT2A |
| RASA1 | BCAR1 |  | NOLC1 | G3BP1 |
| NCK2 | RASA2 |  | AMD1 | CUL3 |
| KIF3C | CLIP2 |  | NCL | RPS6KA5 |
| OPHN1 | WASF1 |  | CUL5 | PBK |
| PLEKHG2 | VCL |  | TFDP1 | CDC6 |
| CEP192 | CDK5RAP2 |  | SLC7A5 |  |
| LLGL1 | ABI1 |  | ATF5 |  |
| CKAP5 | ARF6 |  | RAD21 |  |
| RAPGEF5 | TUBGCP3 |  | ODF2 |  |
