# Supplementary material for: Patient-Derived Organoid Serves as a Platform for Personalized Chemotherapy in Advanced Colorectal Cancer Patients
Source: Front Oncol. 2022 Jun 1;12:883437. doi: 10.3389/fonc.2022.883437 (PMC9205170; doi:10.3389/fonc.2022.883437)
Supplement: Supplementary Table 6 — IC50 values of individual compounds and in combination with oxaliplatin (Oxali) in both oxaliplatin-resistnat and -seinsitve PDOs. [file Table_6.docx]

**Supplementary Table S6.** IC_50_ values of individual compounds and in combination with oxaliplatin (Oxali) in both oxaliplatin resistnat and seinsitve PDOs

**Oxaliplatin resistnat PDOs**

| **Pat. ID** | **Oxali** | **Imatinib** | **Oxali + Imatinib (IC20)** | **DAPT** | **Oxali + DAPT (IC20)** | **Irinotecan** | **Oxali + Irinotecan**  **(IC20)** | **MMC** | **Oxali + MMC**  **(IC10-40)** | **MPM** | **Oxali + MPM (IC20)** | **Vorinostat** | **Oxali + Vorinostat (IC20)** | **TSA** | **Oxali + TSA (IC10)** | **Scriptaid** | **Oxali + Scriptaid (IC10-30)** |
| --- | --- | --- | --- | --- | --- | --- | --- | --- | --- | --- | --- | --- | --- | --- | --- | --- | --- |
|  | **IC50 (μM)** | **IC50 (μM)** | **IC50 (μM)** | **IC50 (μM)** | **IC50 (μM)** | **IC50 (μM)** | **IC50 (μM)** | **IC50 (μM)** | **IC50 (μM)** | **IC50 (μM)** | **IC50 (μM)** | **IC50 (μM)** | **IC50 (μM)** | **IC50 (μM)** | **IC50 (μM)** | **IC50 (μM)** | **IC50 (μM)** |
| **113** | **36.28** | **15** | **16.69** | **51.34** | **23.76** | **8.95** | **7.31** | **0.27** | **18.35** | **4.21** | **7.86** | **0.7** | **11.67** | **0.14** | **20.99** | **2.19** | **2.33** |
| **78m** | **57.15** | **15.2** | **11.25** | **90.93** | **20.56** | **5.75** | **5.7** | **0.13** | **12.44** | **3.71** | **4.69** | **0.65** | **3.58** | **0.12** | **7.7** | **1.65** | **1.2** |
| **99** | **40.6** | **16.42** | **39.24** | **56.11** | **76.03** | **0.43** | **64.45** | **0.55** | **16.82** | **36.41** | **92.44** | **0.65** | **77.24** | **0.08** | **13.79** | **1.53** | **0.33** |
| **87** | **18.53** | **25.72** | **55.4** | **64.82** | **83** | **0.33** | **17.7** | **0.08** | **7.94** | **5.82** | **3.51** | **1.89** | **26.52** | **0.09** | **10.95** | **2.02** | **15.21** |
| **33** | **22.16** | **19.66** | **18.02** | **61.07** | **13.78** | **0.95** | **14.69** | **0.04** | **1.5** | **4.39** | **0.94** | **1.19** | **16.66** | **0.12** | **2.61** | **1.82** | **2.01** |
| **50** | **26.88** | **21.86** | **11.51** | **76.37** | **11.22** | **0.99** | **6.92** | **0.12** | **1.28** | **7.48** | **1.09** | **1.18** | **13.43** | **0.14** | **4.78** | **1.61** | **3.13** |
| **75** | **26.07** | **22.34** | **14.19** | **70.18** | **5.23** | **0.16** | **0.13** | **0.11** | **5.3** | **6.91** | **2.74** | **1.24** | **10.58** | **0.09** | **8.72** | **1.28** | **1.61** |
| **76** | **18.4** | **16.68** | **10.23** | **96.31** | **31.62** | **0.13** | **2.7** | **0.04** | **28.15** | **4.65** | **4.05** | **0.54** | **2.18** | **0.16** | **5.71** | **1.43** | **3.62** |

**Oxaliplatin sensitive PDOs**

| **Pat. ID** | **Oxali** | **Imati-nib** | **Oxali + Imatinib (IC20)** | **DAPT** | **Oxali + DAPT (IC20)** | **Irinotecan** | **Oxali + Irinotecan**  **(IC20)** | **MMC** | **Oxali + MMC (IC10-40)** | **MPM** | **Oxali + MPM (IC20)** | **Vorinostat** | **Oxali + Vorinostat (IC20)** | **TSA** | **Oxali + TSA**  **(IC10)** | **Scriptaid** | **Oxali + Scriptaid**  **(IC10-30)** |
| --- | --- | --- | --- | --- | --- | --- | --- | --- | --- | --- | --- | --- | --- | --- | --- | --- | --- |
|  | **IC50 (μM)** | **IC50 (μM)** | **IC50 (μM)** | **IC50 (μM)** | **IC50 (μM)** | **IC50 (μM)** | **IC50 (μM)** | **IC50 (μM)** | **IC50 (μM)** | **IC50 (μM)** | **IC50 (μM)** | **IC50 (μM)** | **IC50 (μM)** | **IC50 (μM)** | **IC50 (μM)** | **IC50 (μM)** | **IC50 (μM)** |
| **52m** | **5.43** | **30.88** | **66.82** | **151.19** | **200.5** | **0.39** | **2.26** | **0.54** | **0.9** | **43.42** | **170.3** | **3.75** | **157.9** | **0.45** | **9.04** | **4.21** | **9.34** |
| **78p** | **2.06** | **33.28** | **6.89** | **184.9** | **254.8** | **0.15** | **21.36** | **0.23** | **33.31** | **49.16** | **11.42** | **1.43** | **511.6** | **0.21** | **9.33** | **1.84** | **3.747** |
| **94** | **2.79** | **33.79** | **14.76** | **119.7** | **61.51** | **0.15** | **22** | **0.35** | **9.44** | **8.35** | **18.06** | **1.53** | **43.07** | **0.29** | **12.1** | **3.61** | **9.22** |
| **117** | **6.48** | **12.83** | **11.55** | **18.86** | **45.35** | **0.11** | **2.61** | **0.63** | **11.42** | **8.4** | **19.74** | **1.8** | **52.42** | **0.53** | **1.13** | **4.38** | **2.43** |
| **90** | **6.44** | **11.54** | **73.90** | **32.45** | 36.45 | **0.02** | **13.00** | **1.26** | **106.50** | **10.32** | **80.29** | **2.38** | **22.85** | **0.68** | **12.34** | **5.12** | **21.84** |
| **52p** | **4.90** | **20.62** | **12.13** | **24.63** | 9.58 | **0.14** | **1.42** | **1.84** | **6.34** | **14.63** | **9.08** | **3.46** | **8.17** | **0.32** | **6.12** | **3.24** | **5.31** |
| **88** | **8.75** | **31.23** | **31.09** | **31.61** | 33.17 | **1.62** | **11.23** | **0.98** | **53.30** | **18.24** | **28.47** | **1.87** | **14.69** | **1.09** | **0.41** | **4.57** | **3.51** |
| **38** | **1.58** | **18.43** | **13.46** | **40.36** | **20.15** | **0.12** | **10.56** | **2.3** | **34.87** | **24.36** | **38.45** | **2.43** | **16.24** | **1.14** | **5.46** | **3.2** | **5.4** |
